# Tolerance, Adaptation, and Cell Response Elicited by *Micromonospora* sp. Facing Tellurite Toxicity: A Biological and Physical-Chemical Characterization

**DOI:** 10.3390/ijms232012631

**Published:** 2022-10-20

**Authors:** Elena Piacenza, Simona Campora, Francesco Carfì Pavia, Delia Francesca Chillura Martino, Vito Armando Laudicina, Rosa Alduina, Raymond Joseph Turner, Davide Zannoni, Alessandro Presentato

**Affiliations:** 1 Department of Biological, Chemical and Pharmaceutical Science and Technologies, University of Palermo, Viale Delle Scienze, Ed. 16, 90128 Palermo, Italy; 2 Department of Engineering, University of Palermo, Viale Delle Scienze, Ed. 8, 90128 Palermo, Italy; 3 Department of Agricultural, Food and Forest Sciences, University of Palermo, Viale Delle Scienze, Ed. 4, 90128 Palermo, Italy; 4 Department of Biological Sciences, University of Calgary, 2500 University Drive NW, Calgary, AB T2N 1N4, Canada; 5 Department of Pharmacy and Biotechnology, University of Bologna, Via Irnerio 42, 40126 Bologna, Italy

**Keywords:** tellurite, bacterial cell membrane, cell morphology changes, fatty acids, FTIR spectroscopy, oxidative stress, heavy metals, multivariate statistical analysis

## Abstract

The intense use of tellurium (Te) in industrial applications, along with the improper disposal of Te-derivatives, is causing their accumulation in the environment, where oxyanion tellurite (TeO_3_^2^^−^) is the most soluble, bioavailable, and toxic Te-species. On the other hand, tellurium is a rare metalloid element whose natural supply will end shortly with possible economic and technological effects. Thus, Te-containing waste represents the source from which Te should be recycled and recovered. Among the explored strategies, the microbial TeO_3_^2^^−^ biotransformation into less toxic Te-species is the most appropriate concerning the circular economy. Actinomycetes are ideal candidates in environmental biotechnology. However, their exploration in TeO_3_^2−^ biotransformation is scarce due to limited knowledge regarding oxyanion microbial processing. Here, this gap was filled by investigating the cell tolerance, adaptation, and response to TeO_3_^2−^ of a *Micromonospora* strain isolated from a metal(loid)-rich environment. To this aim, an integrated biological, physical-chemical, and statistical approach combining physiological and biochemical assays with confocal or scanning electron (SEM) microscopy and Fourier-transform infrared spectroscopy in attenuated total reflectance mode (ATR-FTIR) was designed. *Micromonospora* cells exposed to TeO_3_^2−^ under different physiological states revealed a series of striking cell responses, such as cell morphology changes, extracellular polymeric substance production, cell membrane damages and modifications, oxidative stress burst, protein aggregation and phosphorylation, and superoxide dismutase induction. These results highlight this *Micromonospora* strain as an asset for biotechnological purposes.

## 1. Introduction

Tellurium (Te) is a metalloid and rare element (1–5 ppm estimated abundance) belonging to the chalcogen group (XVI) and *p*-block of the periodic table that is naturally present in association with minerals, rocks, or sediments [1]. For quite some time, Te has been a neglected element compared to other metals and metalloids, but, in recent years, its exploitation increased drastically [2]. Indeed, the unique physical-chemical properties of Te per se, Te-containing compounds, and Te-based nanomaterials guarantee their broad application in metallurgy, material science, and chemical and electronic industries, to name a few [2]. Te is an energy-critical element as, in its association with cadmium (CdTe), it is involved in producing photovoltaic thin-film solar cells fundamental to generating renewable energy [3,4]. The current use and improper disposal of Te and its derivatives, alongside the increasing worldwide demand for renewable energy, is causing—and will continue to cause—the building-up of these compounds and materials in the environment as toxic and hazardous waste [2,5]. Specifically, Te-containing compounds can leak from smelters, leach (i.e., solubilization and dissolution under acidic or alkaline conditions) from tailing piles, and more from landfills nearby processing facilities across the trophic chain [2]. The most toxic Te chemical species in the environment are the highly soluble and bioavailable oxyanions tellurate (TeO_4_^2−^) and tellurite (TeO_3_^2−^), which threaten human health and ecosystem fitness [6]. Additionally, recent projections highlighted that Te demand will exceed its supply by 2029 [7], posing economic concerns that could determine energy and technological crises. Thus, it is imperative to recover and recycle this element from the environment. CdTe photovoltaic thin-film solar cells represent a large reservoir for Te, yet suitable processes for its recovery are still under study and implementation [2]. However, most of these strategies are multi-step procedures that involve toxic compounds and additives and, more importantly, generate toxic Te oxyanions [2], which can seep into the environment, contributing to the contamination of ecological niches.

According to the concept of the circular economy, the biological reduction by microorganisms of Te oxyanions into the less toxic and bioavailable elemental Te (Te^0^) represents a green and economically effective alternative to chemical and physical approaches [2]. Most of this field of research has been focused on the bacterial transformation of TeO_3_^2−^ into Te^0^, as, considering *Escherichia coli* as a reference bacterial strain, this oxyanion is more toxic than TeO_4_^2−^ [6]. The biotechnological added value of biological approaches is the generation by microorganisms of thermodynamically stable Te nanomaterials [6,8]. Nevertheless, Te bioreclamation is still in its infancy, as several parameters need further optimization to ensure an efficient and cost-effective Te recovery. In turn, the optimization of these conditions relies on a proper and exhaustive understanding of the cell response to TeO_3_^2−^ and the mechanism(s) that diverse microorganisms can elicit to tolerate the oxyanion presence; however, these outlines are still less investigated and elucidated.

Among microorganisms often exploited for biotechnological purposes, members of the Actinobacteria phylum feature genome heterogeneity, which results in different physiological and metabolic properties that make them highly tolerant and resistant to metal and metalloid compounds [9]. Indeed, several Actinobacteria are suitable for the bioremediation and bioreclamation of metals and metalloids [9]. Specifically, *Micromonospora* species inhabit environmental niches such as plant rhizospheres, pyrite-polluted soil, and marine sediments as plant growth-promoting rhizobacteria (PGPR), phosphate-solubilizing bacteria (PSB), metal(loid)-detoxifiers, and nanoparticle producers [10,11,12,13]. Thus, the *Micromonospora* genus holds the genetic versatility required to become an asset from a biotechnological perspective. Nevertheless, only a few *Micromonospora* species have been described to date for their ability to tolerate and/or resist metal(loid) compounds, and none report on the handling of TeO_3_^2−^ by bacterial strains belonging to this genus.

In light of these premises, the present study reports on a detailed characterization of a *Micromonospora* strain isolated from a XIX-century Japanese wallpaper rich in metals and metalloids [14] facing different TeO_3_^2−^ concentrations. Specifically, the mechanisms of oxyanion toxicity and cell adaptation, resistance, and recovery were evaluated by exploring two physiological conditions (i.e., exponentially grown cells and those growing in the presence of tellurite) through a biological and physical-chemical approach supported by statistical analysis.

## 2. Results

### 2.1. Micromonospora Exponentially Grown Cells Facing Tellurite

#### 2.1.1. Tellurite Uptake, Thiol Oxidation, and Reactive Oxygen Species Production

**Figure 1:**
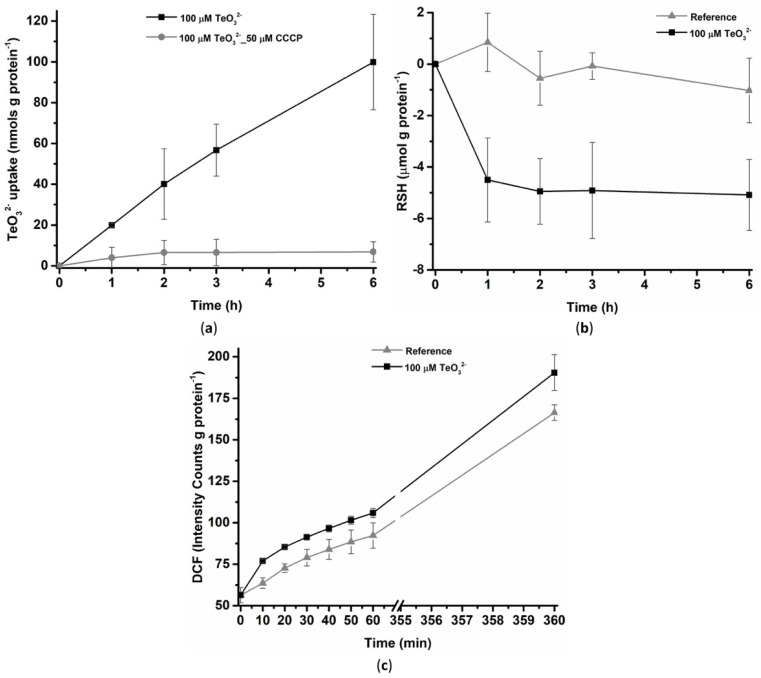
Oxyanion uptake profiles (**a**), evaluation of RSH depletion (**b**), and kinetic of ROS production (**c**) upon exposure of *Micromonospora* exponentially grown cells to 100 μM TeO_3_^2−^. All data points in panel (**c**) referring to ROS produced by TeO_3_^2−^-challenged cells are significantly different (*p* < 0.05) as compared to unchallenged cells.

*Micromonospora* exponentially grown cells (Section 4.1) proficiently handled 100 μM TeO_3_^2−^ toxicity, as any difference in the total protein content of these cells than those unchallenged was not observed during the 6-h incubation (Figure S1a). Bacterial cells exposed to 100 μM TeO_3_^2−^ showed substantial removal of the oxyanion, which constantly increased over time, reaching its maximum value of ca. 100 nmol g protein^−1^ of TeO_3_^2−^ at 6-h (Figure 1a). To evaluate whether TeO_3_^2−^ uptake was driven by the membrane potential [15], the effect of the protonophore and electron-transfer uncoupler carbonyl-cyanide m-chlorophenylhydrazone (CCCP) on the oxyanion transport within bacterial cells, was evaluated. As a result, CCCP addition (50 μM) led to a drastic decrease in TeO_3_^2−^ removal by bacterial cells that plateaued from 2-h of incubation onwards. A low amount of oxyanions (ca. 6 nmol g protein^−1^) was taken up by bacterial cells upon incubation with CCCP. This outline is likely consistent with either sorption of TeO_3_^2−^ occurring at the cell surface or incomplete accessibility of the CCCP to the entire cell population. Additionally, the difference observed in oxyanion removal by *Micromonospora* exponentially grown cells exposed to 100 μM TeO_3_^2−^ in the absence or presence of CCCP indicated that ca. 90 nmol g protein^−1^ of TeO_3_^2−^ entered the cells within 6-h of incubation. Once TeO_3_^2−^ reached the intracellular milieu of *Micromonospora* cells, it led to a loss of the thiol (RSH) pool (Figure 1b). The effect of such an oxidized intracellular environment was also reflected by the increased level of intracellular reactive oxygen species (ROS)—detected by the oxidative stress-sensitive probe 2′,7′-dichlorofluorescein diacetate (DCF)—starting from 10 min up to 1-h exposure to TeO_3_^2−^ that perdured till 6-h of incubation (Figure 1c) as compared to unchallenged cells. These effects highlight a clear situation of intracellular stress deriving from oxyanions’ entry and their subsequent processing.

#### 2.1.2. Effect of Tellurite on the Membrane Potential

**Figure 2:**
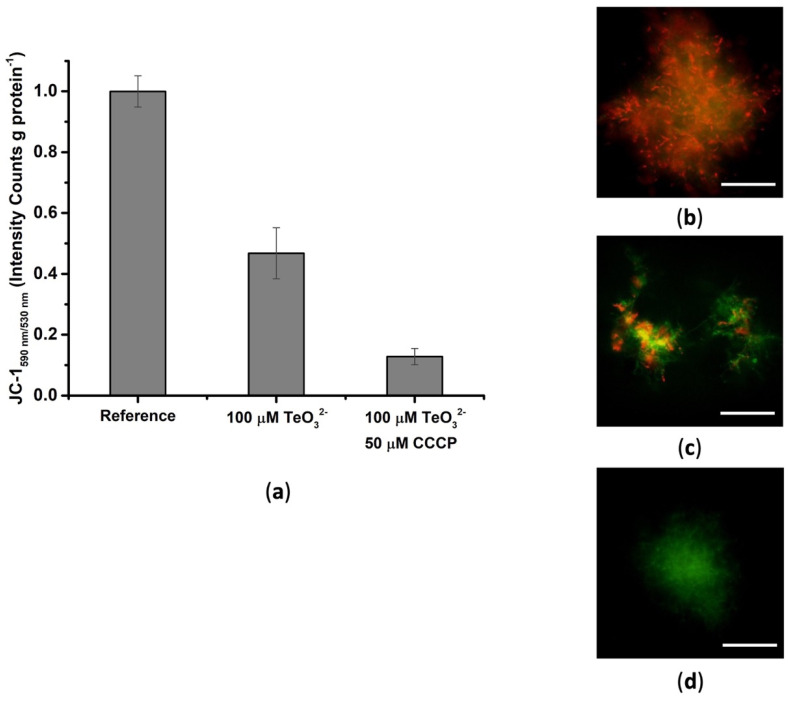
Effect of tellurite on the membrane potential of *Micromonospora* exponentially grown cells, evaluating the 590/530 JC-1 fluorescence emission ratios (**a**) and fluorescence imaging of (**b**) unchallenged cells or those exposed to (**c**) 100 μM TeO_3_^2−^ and (**d**) 50 μM CCCP. Scale bar 50 μm.

The cationic dye 5,5′,6,6′-tetrachloro-1,1′,3,3′-tetraethylbenzimidazolylcarbocyanine iodide (JC-1) is membrane-permeant, and it can be used as an indicator of the electrical component (Δψ) of membrane potential [16]. Here, we used the JC-1 to assess whether the physiological membrane potential of *Micromonospora* cells was affected by exposure to TeO_3_^2−^. Specifically, depending on the membrane potential, the JC-1 dye undergoes a variation in its conformation and, therefore, fluorescence emission, shifting from red (dye aggregates) to green (dye monomers) at high and low membrane potential values, respectively [17]. Unchallenged *Micromonospora* cells displayed both a high 590 (red)/530 (green) emission ratio and a diffuse red fluorescence (Figure 2a,b), which can be ascribed to healthy and viable cells. Conversely, bacterial exposure to 100 μM TeO_3_^2−^ caused a partial decrease in the 590/530 emission ratio (Figure 2a,c). This result indicates that TeO_3_^2−^ uptake does not induce a total depolarization of the membrane potential as vice versa observed with CCCP (Figure 2a,d). Therefore, as is also shown in other bacterial systems [15], TeO_3_^2−^ uptake in *Micromonospora* cells is likely driven by the ΔpH component of the proton motive force (pmf).

#### 2.1.3. Tellurite Effect on the Fatty Acid Profile

Considering the ability of *Micromonospora* exponentially grown cells in processing 100 μM TeO_3_^2−^, the influence of this oxyanion on the whole-cell fatty acids was assessed by incubating bacterial cells to a high TeO_3_^2−^ concentration (5 mM; Table 1). As a result, challenged cells featured a slight increase in saturated fatty acid content. Specifically, there was an evident shift from odd-numbered fatty acids toward even-numbered ones compared to control cells. Concomitantly, the relative percentage abundance of unsaturated fatty acids decreased. This effect was due to the low content of the monounsaturated fatty acid C_17:1_, whereas the saturated C_18:0_ and the branched *iso-*C_16:0_ ones significantly increased; indeed, the latter doubled their content in TeO_3_^2−^-exposed cells. Additionally, *iso-*C_15:0_ and *iso-*C_17:0_ slightly increased in challenged cells compared to unchallenged ones. Thus, this evidence highlights how *Micromonospora* exponentially grown cells responded to the stress exerted by a high concentration of tellurite by increasing their membrane rigidity.

### 2.2. Micromonospora Cells Growing in the Presence of Different Tellurite Concentrations

#### 2.2.1. Bacterial Tolerance towards TeO_3_^2−^

**Figure 3:**
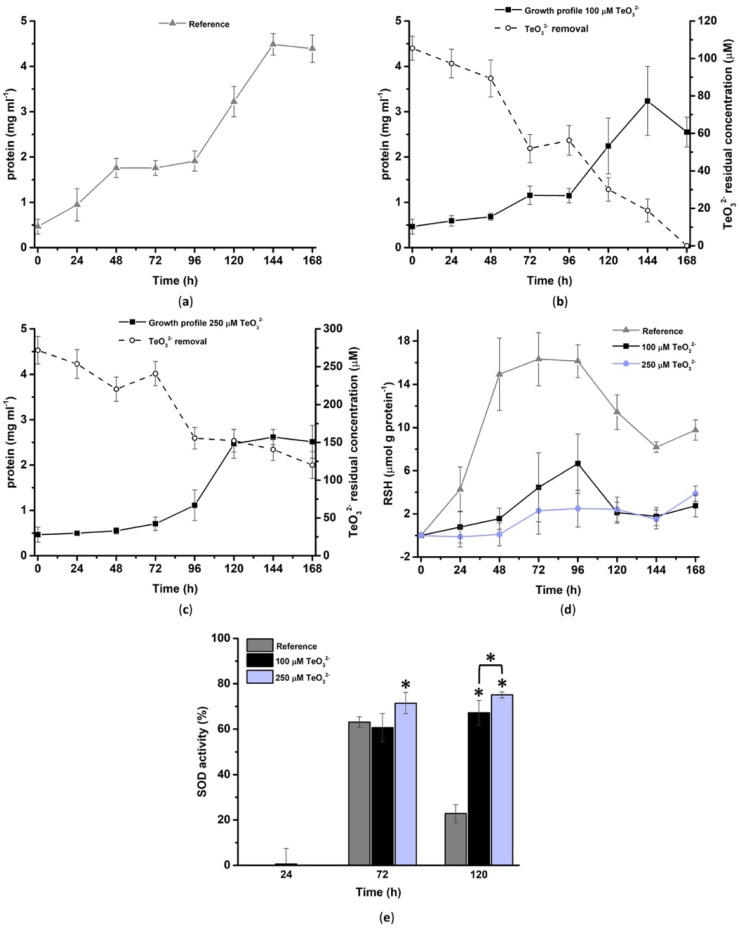
Growth profiles of *Micromonospora* unchallenged cells (**a**) and challenged ones with either 100 (**b**) or 250 μM TeO_3_^2−^ (**c**). The oxyanion consumption by *Micromonospora* cells is displayed on the secondary y-axes in (**b**) and (**c**). Evaluation of the loss of reduced thiol (RSH) content (**d**) and superoxide dismutase activity (**e**) as a stress response to TeO_3_^2−^ elicited by *Micromonospora* growing cells (* *p* < 0.05).

Unchallenged *Micromonospora* cells displayed the typical growth profile of most actinomycetes (Figure 3a). Indeed, a first rapid growth (RG1) phase was observed up to 48-h of incubation, followed by a transition (T) phase between 48 and 96-h, a subsequent second RG phase (RG2) from 96 to 144-h, and, finally, a stationary phase (144–168-h). On the other hand, both 100 and 250 μM TeO_3_^2−^ negatively affected the growth of the *Micromonospora* strain, as indicated by the lower biomass yield observed over the timeframe considered (Figure 3b,c). Regardless of the initial oxyanion concentration, this actinomycete highlighted a prolonged lag phase of either 48 or 72-h upon bacterial incubation with 100 or 250 μM TeO_3_^2−^, respectively. Nevertheless, the growth profile of *Micromonospora* cells exposed to 100 μM TeO_3_^2−^ better resembled that of the unchallenged ones since RG1, T, and RG2 phases were still detected. However, 100 μM TeO_3_^2−^ caused death events at the last stage (168-h) of bacterial growth, resulting in a biomass yield comparable to cells grown in the presence of 250 μM TeO_3_^2−^. Additionally, 500 μM TeO_3_^2−^ triggered a more drastic effect on *Micromonospora* cells, as no active bacterial growth was detected (Figure S1b). TeO_3_^2−^ removal by *Micromonospora* cells mirrored the growth profiles. Then, 100 μM TeO_3_^2−^ was removed within 168-h of bacterial growth, while 250 μM TeO_3_^2−^ was partially converted (ca. 130 μM), highlighting that this bacterial strain can bioprocess a threshold tellurite concentration. Moreover, bacterial cells did not actively remove 500 μM TeO_3_^2−^, confirming the cytotoxic effect of oxyanions toward the *Micromonospora* strain at this concentration (Figure S1b).

Trends of the intracellular RSHs pool resembled the observed growth profiles up to 72-h (250 μM TeO_3_^2−^) and 96-h (control and 100 μM TeO_3_^2−^) of bacterial incubation (Figure 3d). The moment unchallenged cells or those growing in the presence of 100 μM TeO_3_^2−^ entered their RG2 phase (96–144-h), RSHs decreased, although it was more drastic in the case of tellurite-growing cells. Instead, bacterial cells experiencing the presence of 250 μM TeO_3_^2−^ displayed a low and constant amount of RSHs from 72 to 144-h, despite the exponential growth phase occurring between 72 and 120-h. The overall lower content of RSHs in challenged cells suggests that TeO_3_^2−^ strongly compromises these important buffering molecules, which, upon their oxidation, generate oxidative damage. Nevertheless, the intracellular RSH pool slightly increased at the 168-h time point for all conditions tested, highlighting that *Micromonospora* cells can recover from oxidative stress, likely involving other enzymatic systems. In this regard, the superoxide dismutase (SOD) activity assay (Figure 3e) revealed that at the earliest stage (24-h) of bacterial growth, cells featured a low enzymatic activity, while it increased similarly up to 72-h incubation, although only in the case of 250 μM TeO_3_^2−^-grown cells, the SOD activity increase resulted as statistically significant with the respect of unchallenged cells. Finally, at 120-h of bacterial growth, cells facing the TeO_3_^2−^ challenge still featured an enhanced SOD activity, which dropped down in the case of cells not experiencing oxyanions’ stress, further reinforcing the occurrence of an oxidized intracellular environment that needs to be restored.

**Figure 4:**
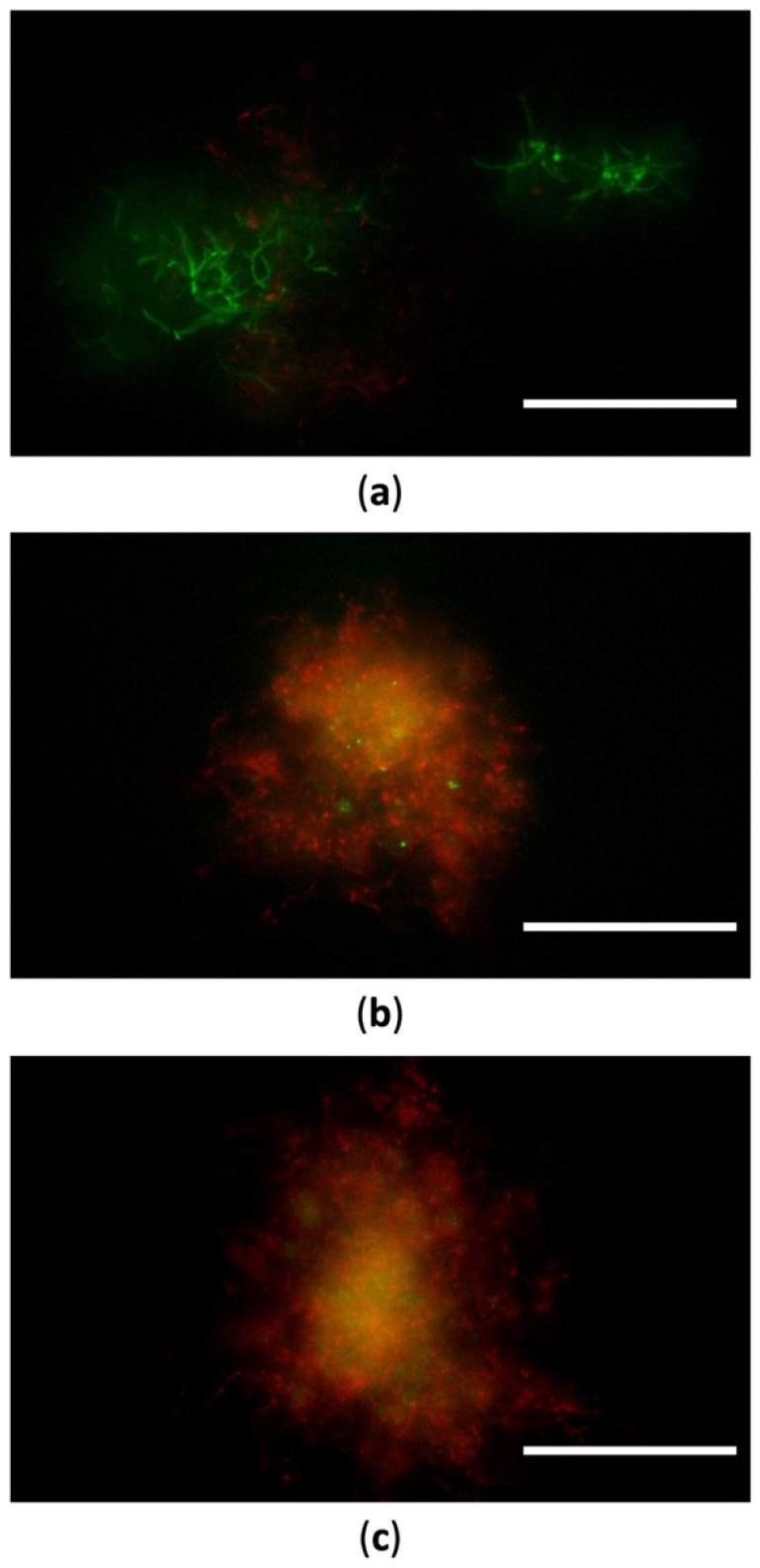
Fluorescence microscopy of *Micromonospora* unchallenged cells (**a**) and those grown in the presence of either 100 (**b**) or 250 μM TeO_3_^2−^ (**c**) for 24-h. Scale bar 50 μm.

In line with growth profiles, a cell viability assay (Section 4.4), carried out through fluorescence microscopy, revealed a strong green-fluorescent signal only in the case of *Micromonospora* unchallenged cells at its RG1 phase (Figure 4a). On the contrary, the lag phase determined by the TeO_3_^2−^ challenge led to the detection of a mixed population of viable and non-viable cells, as indicated by the simultaneous green, red, and yellow fluorescence emission (Figure 4b,c). This evidence suggests that propidium iodide (red dye) can penetrate cells featuring damage to their cell membranes.

#### 2.2.2. Morphological Characterization of *Micromonospora* Cells under Tellurite Stress

**Figure 5:**
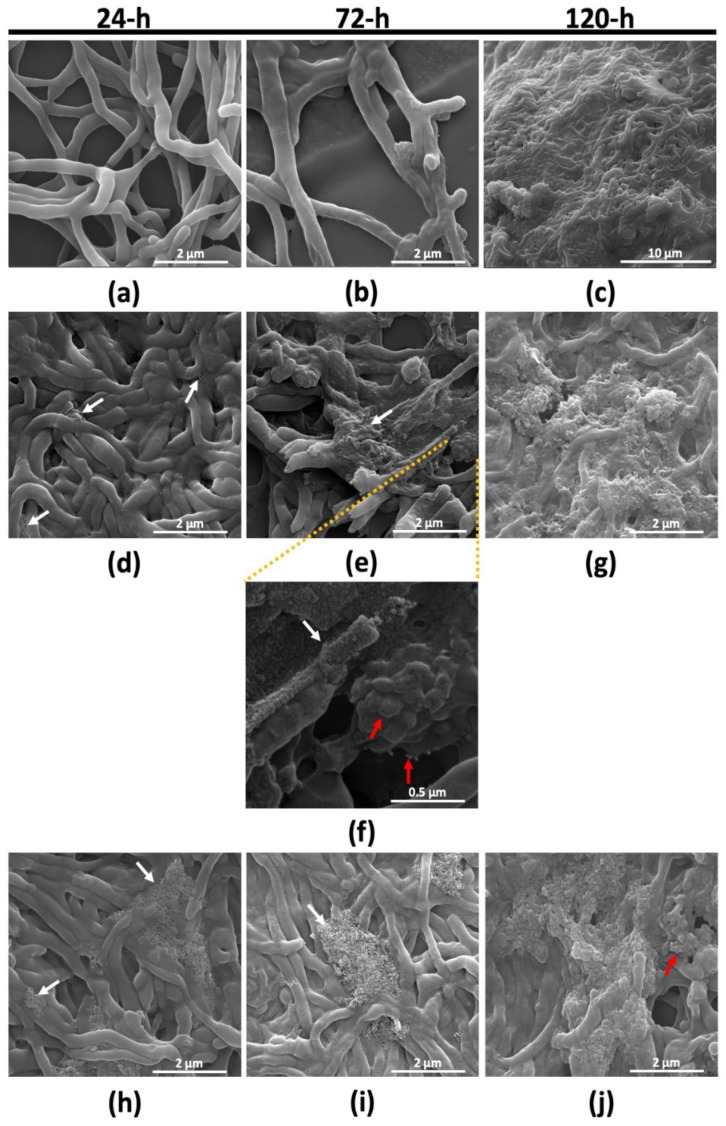
SEM micrographs depicting *Micromonospora* unchallenged cells (**a**–**c**) and those grown in the presence of either 100 (**d**–**g**) or 250 μM TeO_3_^2−^ (**h**–**j**) over time (24, 72, and 120-h). White arrows highlight oxyanion biosorption on the bacterial surface, while the appearance of membrane vesicle-like structures is indicated with red arrows.

*Micromonospora* cells growing in the presence of either 100 or 250 μM TeO_3_^2−^ were imaged through scanning electron microscopy (SEM) to highlight morphological changes in response to the stress exerted by oxyanions and depending on the incubation time considered (Figure 5). At 24-h growth, unchallenged cells highlighted loosely packed hyphae, which appeared to have a smooth and clean texture at the surface, lacking any superficial defect (Figure 5a). Afterward, bacterial hyphae did not highlight an orthodox morphology (Figure 5b) as that observed for cells in the RG1 phase, most likely due to cells’ entry in their transition phase (72-h), where an actual arrest of the bacterial growth occurred (Figure 3a). Finally, *Micromonospora* cells reaching the RG2 phase (120-h) formed a dense network of hyphae (Figure 5c), which macroscopically corresponded to the appearance of cell floccules in the flask. On the contrary, bacterial cells facing the oxyanion challenge displayed more swollen hyphae tightly packed to one another, forming clumps of aggregated mycelium with a certain degree of superficial roughness (Figure 5d,h). This aspect was consistent with the appearance of cell floccules at the earliest stage of bacterial growth. Additionally, bacterial hyphae produced extracellular material, likely containing oxyanions, adsorbed on the cell surface (indicated by white arrows). This sorption event was more evident for *Micromonospora* cells growing in the presence of 250 μM TeO_3_^2−^ at the beginning of the bacterial incubation (Figure 5h), although this aspect occurred for each incubation time considered, and its extent was exasperated as a function of both time and concentration of oxyanions supplied (Figure 5d–j). Moreover, TeO_3_^2−^-grown cells featured the rising of both small and large vesicle-like structures (indicated by the red arrow; Figure 5f,j). The delayed emergence of such a structure for cells grown in the presence of 250 μM TeO_3_^2−^ could be ascribed to the extensive lag phase observed (Figure 3c).

#### 2.2.3. Fourier Transform Infrared Spectroscopy in Attenuated Total Reflectance (ATR-FTIR) Mode

**Figure 6:**
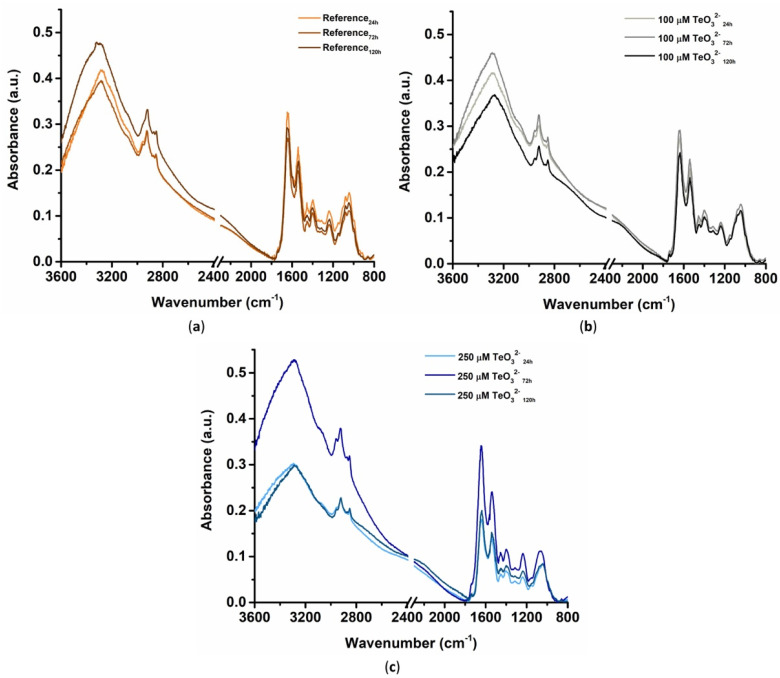
ATR-FTIR spectra of *Micromonospora* cells grown for 24, 72, and 120-h in the absence (**a**) or presence of (**b**) 100 μM or (**c**) 250 μM TeO_3_^2−^.

ATR-FTIR spectroscopy highlighted vibrational modes typical of lipids, proteins, polysaccharides, and nucleic acids for both unchallenged cells and those incubated with TeO_3_^2−^ over time (Figure 6). Full band assignments are reported in Table S1.

The presence of lipids was mainly determined from IR contributions in the 2960–2850 cm^−1^ region and the weak absorption detected at ca. 1740 cm^−1^ (Figure 6; Table S1). These signals correspond to -CHx stretching (asymmetric and symmetric) vibrations within aliphatic chains of fatty acids (2960–2850 cm^−1^) and the stretching of carbonyl (-C=O; 1740 cm^−1^) of bacterial lipids and triglycerides [18,19]. Although all samples showed these IR contributions, the exposure of *Micromonospora* cells to TeO_3_^2−^ determined variations within the 2960–2850 cm^−1^ region. Indeed, both the asymmetric -CH_3_ (ca. 2950 cm^−1^) and the symmetric -CH_2_ stretching (ca. 2850 cm^−1^) vibrations shifted to bigger wavenumbers upon bacterial incubation with the oxyanion (Table S1). This phenomenon was more evident in *Micromonospora* cells grown in the presence of 250 μM TeO_3_^2−^, as the asymmetric -CH_3_ stretching vibration shifted from ca. 2953 cm^−1^ (untreated cells) to 2962–2960 cm^−1^ (Table S1). Similarly, ATR-FTIR spectra of 100 μM TeO_3_^2−^_72h_ and 250 μM TeO_3_^2−^_72h_ showed asymmetric -CH_2_ stretching vibrations at bigger wavenumbers (2926 cm^−1^) than TeO_3_^2−^-free cells (Table S1). Moreover, cells grown in the presence of both TeO_3_^2−^ concentrations featured, over time, similar normalized FTIR integrals referring to -CH_x_ signals, which were different from those calculated for unchallenged cells (Figure S2). Insights regarding the potential effect of TeO_3_^2−^ on the bacterial membrane were gathered by evaluating the ratios between asymmetric -CH_2_ stretching vibration and either asymmetric -CH_3_ stretching (A_νas (CH2)_/A_νas (CH3)_) or symmetric -CH_2_ (A_νas (CH2)_/A_νs (CH2)_) stretching vibrations (Figure S3). Overall, the addition of TeO_3_^2−^ led to increased integral ratios from 72-h onwards, indicating a higher degree of lipid saturation (A_νas (CH2)_/A_νas (CH3)_) [20,21] and a lower disorder in lipid acyl chains (A_νas (CH2)_/A_νs (CH2)_) [22] than in unchallenged cells (Figure S3). At 72-h of bacterial growth, the change of lipid saturation was more significant upon incubation with 100 μM TeO_3_^2−^ than in the other conditions tested (Figure S3).

All samples showed IR contributions typical of proteins, namely, amide A (3271–3290 cm^−1^), amide B (3061–3073 cm^−1^), amide I (1686–1617 cm^−1^), amide II (1534–1558 cm^−1^), and amide III (1237–1248 cm^−1^) bands (Figure 6; Table S1). However, TeO_3_^2−^ presence caused differences in the shifting, appearance, and disappearance of these IR signals (Table S1), which were highlighted by spectral deconvolutions in the 1780–1480 cm^−1^ region (Figure S4; Tables S2–S4). Although vibrational modes deriving from α-helix secondary structure (1660–1650 cm^−1^) were detected for all samples, IR absorption bands centered at ca. 1690–1680 cm^−1^, 1670 cm^−1^, and 1640–1610 cm^−1^ were attributed to β-antiparallel, β-turn, and β-sheet structures, respectively, while random coil protein bands were observed at 1650–1640 cm^−1^ (Figure S4b,c; Tables S3 and S4). Based on the normalized integrals obtained for amide I bands, an estimation (as a relative percentage) of the proteins’ secondary structure was determined (Figure S5). TeO_3_^2−^ caused the appearance of IR contributions typical of β-strand structures at 24-h of bacterial growth (Figure S5b,c; Tables S3 and S4). Indeed, a higher β-sheet contribution (ca. 65 and 67%) was detected in ATR-FTIR spectra of 100 μM TeO_3_^2−^_24h_ and 250μM TeO_3_^2−^_24h_ than Reference_24h_, which featured a larger amount of α-helix structure (ca. 64%) (Figure S5). Additionally, a high percentage of random coil proteins, alongside the appearance of β-antiparallel structures, were calculated for both 100 μM TeO_3_^2−^_24h_ and 250 μM TeO_3_^2−^_72h_ (Figure S5b,c). Furthermore, 250 μM TeO_3_^2−^_24h_ showed instead only α-helix (ca. 33%) and β-sheets (ca. 67%) secondary structure (Figure S5b,c). However, TeO_3_^2−^-free and challenged cells featured similar protein secondary structure at 120-h of growth, β-sheet contributions being the most represented (Figure S5). Similar to the amide I band, the IR signals attributable to amide II (1560–1530 cm^−1^) underwent modifications upon bacterial exposure to the oxyanion (Tables S3 and S4). All ATR-FTIR spectra featured the typical amide II band centered at ca. 1545 cm^−1^, yet TeO_3_^2−^ stress led to the appearance of other three IR contributions (ca. 1560, 1535, and 1520 cm^−1^) deriving from the same vibrational mode. These additional signals were only partially present for unchallenged cells in Reference_72h_ (Tables S2–S4). This phenomenon was emphasized for 250 μM TeO_3_^2−^_24h_ and 250 μM TeO_3_^2−^_120h_, which showed four and three contributions related to the amide II bands, respectively (Table S4). The oxyanion also influenced the abundance of these IR contributions (as normalized integrals) (Figure S6). The 1545 cm^−1^ and 1525 cm^−1^ amide II bands featured comparable integrals in the ATR-FTIR spectra, although the former were drastically reduced after 72-h of bacterial growth under all conditions tested (Figure S6). The 1525 cm^−1^ contribution was instead only observed for Reference_72h_, 100 μM TeO_3_^2−^_72h_, and 250 μM TeO_3_^2−^_24h_ (Figure S6). Conversely, 100 μM TeO_3_^2−^_120h_ and 250 μM TeO_3_^2−^_72h_ displayed the highest normalized integrals referring to the 1535 cm^−1^ amide II band, while that centered at ca. 1560 cm^−1^ was detected only for cells grown for 24 and 120-h in the presence of 250 μM oxyanion (Figure S6b,c). Further insights regarding the membranes’ fluidity and protein content were obtained by evaluating the ratio between the normalized integrals referring to the amide II band and the asymmetric -CH_3_ stretching vibration (A_amide II_/A_νas (CH3)_) typical of cellular lipids (Figure S6d). TeO_3_^2−^ exerted a concentration-dependent effect on these IR contributions and, in turn, membrane fluidity. Indeed, 250 μM TeO_3_^2−^_24h_ displayed a lower A_amide II_/A_νas (CH3)_ ratio, hence, membrane fluidification, than unchallenged samples after 24-h of growth, while a higher ratio (membrane rigidification) was obtained for 100 μM TeO_3_^2−^_72h_ than Reference_72h_ (Figure S6d). After 120-h of growth, ATR-FTIR spectra of cells incubated with both oxyanion concentrations showed rigidified membranes, as indicated by the higher A_amide II_/A_νas (CH3)_ ratio than unchallenged cells (Figure S6d).

A second deconvolution was performed in the 1500–980 cm^−1^ region of ATR-FTIR spectra (Figure S7; Tables S5–S7) to better highlight contributions deriving from diverse macromolecules. The IR absorption between 1430 and 1360 cm^−1^ can be attributed—in addition to vibrational modes of some polysaccharides, lipids, and proteins—to symmetric -COO^−^ stretching vibrations of cellular peroxidation products [23,24]. Samples of 100 μM TeO_3_^2−^_24h_, 100 μM TeO_3_^2−^_120h_, and 250 μM TeO_3_^2−^_120h_ displayed higher normalized integrals referring to this IR absorption than unchallenged cells (Figure S8a). No variations (*p* > 0.05) were instead observed at 72-h of growth, regardless of the addition of TeO_3_^2−^ (Figure S8a). Moreover, the oxyanion stress triggered both the shift of the 1310–1313 cm^−1^ band related to -C-(OH) stretching towards 1302–1306 cm^−1^ and the appearance of additional IR contributions in the 1290–1310 cm^−1^ region (Figure S7b,c; Tables S6 and S7). The latter IR variations can be related to the -C-OH stretching of acetic acid molecules within bacterial cells because of peroxidation processes [23]. IR signals centered at ca. 1235 cm^−1^ can be associated with -C-O-P stretching vibrations deriving from phosphorylated proteins [25]. Based on the estimation of the ratio between the normalized integrals of this contribution and that of the amide I band (A_ν (C-O-P)_/A_amide I_), *Micromonospora* cells grown in the presence of TeO_3_^2−^ featured a greater extent of phosphorylated proteins than oxyanion-free cells (Figure S8b). In the case of cells facing the lowest TeO_3_^2−^ concentration, the highest phosphorylation was observed after 24-h of bacterial growth, which tended to decrease over time and became comparable to unchallenged cells (Figure S8b). Conversely, protein phosphorylation increased in a time-dependent fashion upon 250 μM TeO_3_^2−^ supply to the growth medium, being the highest for 250 μM TeO_3_^2−^_72h_ and 250 μM TeO_3_^2−^_120h_ (Figure S8b), indicating a concentration-dependent effect of this oxyanion on the phosphorylation of proteins.

IR contributions identified by spectral deconvolutions in the 1200–950 cm^−1^ region were mostly attributed to polysaccharides (Figure S7; Tables S5–S7), as these absorption bands are typical of α_(1,3)_, α_(1,4)_, β_(1,3)_, and β_(1,4)_ glycosidic bonds or other polysaccharide-related vibrations [26]. All samples featured polysaccharide bands centered at ca. 1150, 1104, 1079, and 1040 cm^−1^, yet shifts towards bigger wavenumbers were detected upon TeO_3_^2−^ stress (Tables S5–S7). A downshift was observed only for the 1104 cm^−1^ band in 250 μM TeO_3_^2−^_72h_, which featured a contribution at ca. 1095 cm^−1^ (Table S7). Moreover, ATR-FTIR spectra collected for *Micromonospora* cells grown for 24 or 72-h in the presence of 250 μM TeO_3_^2−^ did not display the IR signal centered at ca. 1079 cm^−1^ (Table S7). The oxyanion presence led also to the appearance of additional polysaccharide vibrational modes in 100 μM TeO_3_^2−^_24h_ (ca. 1039 cm^−1^), 100 μM TeO_3_^2−^_72h_ (ca. 1002 cm^−1^), and 250 μM TeO_3_^2−^_120h_ (ca. 1017 cm^−1^; Tables S6 and S7). Additionally, TeO_3_^2−^ determined variations in the abundance of polysaccharide IR contributions (Figure S9). Although all ATR-FTIR spectra showed integrals related to 1104 and 1040 cm^−1^ bands as the most represented over time, changes in their distribution were observed (Figure S9). These differences were more evident for 100 μM TeO_3_^2−^_24h_ and 250 μM TeO_3_^2−^_120h_, as these samples highlighted low IR absorption attributable to polysaccharides and comparable 1104 and 1040 cm^−1^ integrals (Figure S9b,c). Moreover, lower (100 μM TeO_3_^2−^_120h_ and 250 μM TeO_3_^2−^_120h_) or higher (250 μM TeO_3_^2−^_72h_) areas for the 1104 cm^−1^ signal were obtained in TeO_3_^2−^-incubated cells than those unchallenged (Figure S9). The 1040 cm^−1^ integrals varied as a function of the oxyanion concentration in the culture broth (Figure S9b,c). When 100 μM TeO_3_^2−^ was used, these integrals increased over time, while an opposite trend was observed upon the addition of 250 μM TeO_3_^2−^ (Figure S9b,c). Overall, integrals referring to the 1150 cm^−1^ band were similar, while the highest variability was obtained for those derived from the 1079 cm^−1^ signal (Figure S9). Indeed, these integrals decreased over time for unchallenged cells, while those exposed to both TeO_3_^2−^ concentrations featured high 1079 cm^−1^ contributions at 120-h of growth (Figure S9). Finally, IR absorption bands related to thiol (-SH) -containing or -deriving molecules [27] were detected in the 1520–780 cm^−1^ region of ATR-FTIR spectra (Table S1).

#### 2.2.4. Multivariate Statistical Analysis

**Figure 7:**
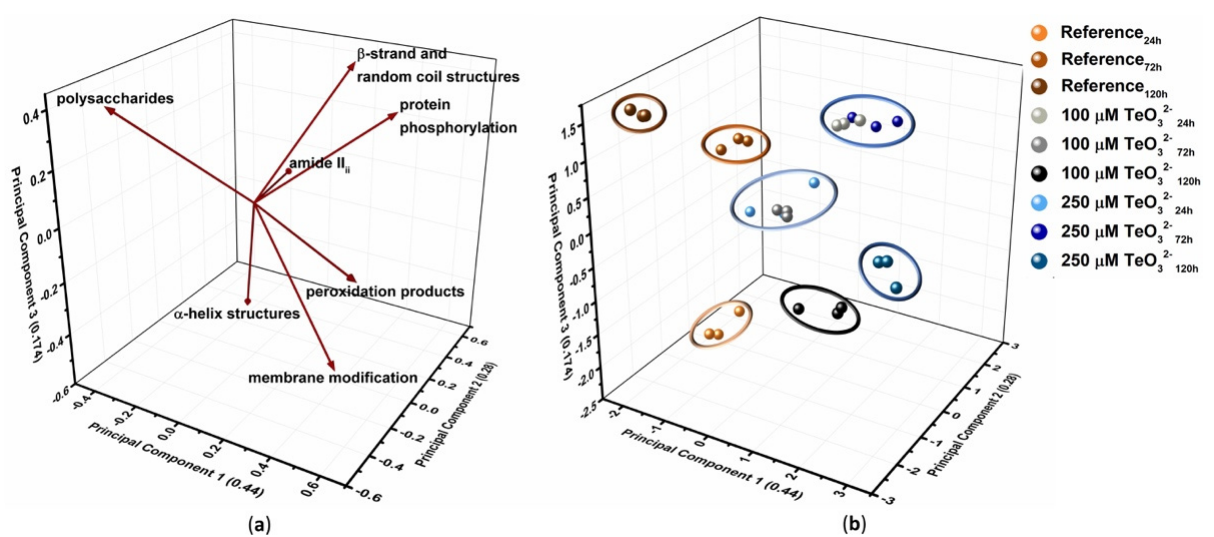
Representation of (**a**) loading and (**b**) score plots obtained by PCA performed on IR vibrational modes contributing the most to sample variability. Clusters identified by PCA are highlighted in (**b**) by colored circles.

Principal Component Analysis (PCA) was performed on the collected ATR-FTIR spectra to overall assess macromolecules involved in TeO_3_^2−^ bioprocessing and their changes (Figure 7). Based on the ATR-FTIR results, IR contributions and ratios that described the stress exerted by TeO_3_^2−^ onto *Micromonospora* cells were chosen as variables.

PCA accounted for 89.4% of the original information of the variables and highlighted three main PCs whose parameters (i.e., percentage of original information and discriminating variable vectors) are reported in Table 2.

Vectors describing amide II_II_, β-strand and random coil structures, and protein phosphorylation strongly correlated in the 3-dimensional (3D) subspace of PCA (Figure 7a). A similar linear relationship was also observed for vectors related to membrane modification and peroxidation products (Figure 7a). The PCA score plot showed the grouping of samples within seven clusters, which mostly coincide with the analysis in triplicate of the samples (Figure 7b). Nevertheless, two major clusters containing the triplicates of (i) 100 μM TeO_3_^2−^_24h_ and 250 μM TeO_3_^2−^_72h_ (cluster 4) and (ii) 100 μM TeO_3_^2−^_72h_ and 250 μM TeO_3_^2−^_24h_ (cluster 5) were identified (Figure 7b). PCA separated unchallenged cells for their IR contributions deriving from polysaccharides and α-helix structures (Figure 7). The former were discriminating for clusters 2 (Reference_72h_) and 3 (Reference_120h_), while the higher amount of α-helix secondary structure of Reference_24h_ than the other samples determined the isolation of cluster 1 in the 3D subspace Figure 7 and Figure S5). Triplicates of 100 μM TeO_3_^2−^_24h_ and 250 μM TeO_3_^2−^_72h_ were grouped in cluster 4 for their similar and elevated contributions deriving from β-strand and random coil contents (as relative percentages) and phosphorylated proteins (as A_ν (C-O-P)_/A_amide I_ ratios) (Figure 7, Figures S5 and S8). The latter variable, alongside the modification of the amide II band (i.e., amide II_II_), also influenced the distribution of 100 μM TeO_3_^2−^_72h_ and 250 μM TeO_3_^2−^_24h_, which clustered together (Figure 7). The high IR signals referring to cell membrane integrity and the appearance of peroxidation products played a crucial role for clusters 6 (100 μM TeO_3_^2−^_120h_) and 7 (250 μM TeO_3_^2−^_120h_). However, the higher extent of cell membrane rigidification and the lower level of peroxidation products observed for 100 μM TeO_3_^2−^_120h_ than 250 μM TeO_3_^2−^_120h_ determined the separation of these samples within two clusters (Figure 7, Figures S2, S3, S6 and S8a). Moreover, 250 μM TeO_3_^2−^_120h_ was influenced by an elevated A_ν (C-O-P)_/A_amide I_ ratio (Figure 7and Figure S8b).

## 3. Discussion

Few members of actinomycetes are known for their ability to cope with the toxicity exerted by TeO_3_^2−^ [28,29,30,31,32]. However, a detailed understanding of the cell response—and the oxyanion bioprocessing strategies—elicited by these bacterial strains to counteract TeO_3_^2−^ still represents a gap in this field of research. The capability of *Micromonospora* cells to face TeO_3_^2−^ toxicity fits well with the biotechnological potential of this genus, and it links to its metabolic versatility.

### 3.1. TeO_3_^2−^ Effects on Exponentially Grown Cells

The toxicity of TeO_3_^2−^ preponderantly derives from its entrance within *Micromonospora* exponentially grown cells (Figure 1a). Here, the strong repression of the oxyanion uptake that follows the addition of the electron uncoupler CCCP (Figure 1a), alongside the partial depolarization effect of TeO_3_^2−^ on the *Micromonospora* cell membrane (Figure 2a,c), suggests that the oxyanion entry mainly occurs through a ΔpH-dependent mechanism. As shown in other bacterial systems, this transport depends on low- (Pit family) or high- (Pst family) affinity phosphate transporters, as well as monocarboxylate ones (i.e., acetate permease—ActP—transporters) [8]. Pit- and ActP-mediated uptakes are ΔpH-dependent processes [15,33,34,35,36], while the Pst system relies on ATP hydrolysis [37]. Since most actinomycetes, including *Micromonospora* species, are known as phosphate solubilizing bacteria (PSB) [10,38,39], TeO_3_^2−^ uptake by *Micromonospora* cells is possibly due to low-affinity phosphate transporters.

Once TeO_3_^2−^ enters bacterial cells, it can be bioprocessed through Painter-type reactions, where (i) RSH-containing molecules first interact with the oxyanion generating a relatively stable intermediate (RS-Te-RS), (ii) enzymes (e.g., glutathione or thioredoxin reductases) convert it into the highly unstable RS-Te^−^, and (iii) the latter spontaneously dismutates, producing Te^0^ [6]. The rapid loss of intracellular RSH after 1-h of oxyanion exposure (Figure 1b) suggested the occurrence of a Painter-type reaction in *Micromonospora* exponentially grown cells incubated with TeO_3_^2−^. This observation agrees with several reports evaluating RSH depletion in the first 2.5-h of bacterial exposure to oxyanions [40,41,42], corroborating the role of RSHs as buffering molecules against TeO_3_^2−^. Particularly, in the case of actinomycetes such as *Micromonospora*, mycothiols (MSHs)—highly abundant in this bacterial order and featuring greater redox stability than glutathione (GSH)—could be responsible for this first oxyanion bioprocess step [43]. During Painter-type reactions, the formation of the RS-Te-SR intermediate determines the production of superoxide ions (**^·^**O_2_^−^), which may add to those derived from the decoupling of the electron transport chain potentially mediated by TeO_3_^2−^ [8]. Finally, TeO_3_^2−^ can inhibit heme biosynthesis, inducing an intracellular accumulation of protoporphyrin IX, which can generate ROS by electron or energy transfer [44]. Here, the exposure of *Micromonospora* exponentially grown cells to TeO_3_^2−^ determined a higher production of ROS than unchallenged cells (Figure 1c), further confirming previous observations reported on Gram-negative or -positive microorganisms facing this oxyanion [45,46,47,48,49].

Similar to most xenobiotics, the first target of TeO_3_^2−^ is the microbial cell membrane, which undergoes perturbations that determine the impairment of several physiological functions, such as those performed by energy-transducing systems located at the membrane level [50]. Bacterial strains must respond to these environmental changes by adapting and maintaining a certain degree of membrane integrity to survive. Microorganisms can accomplish this task by altering the fatty acid profile that features membrane phospholipids, eliciting strategies that include: (i) changes in the saturation degree of the fatty acid acyl chain, (ii) alteration of fatty acid branching at either the *iso* or *anteiso* position of the acyl chain, (iii) *cis*-*trans* isomerization of carbon double bonds, (iv) formation of cyclopropane structure in the acyl chain, and (v) production of polyunsaturated fatty acids in response to adverse environmental factors (i.e., temperature, pressure, salinity, and pH) and the presence of organic xenobiotics such as solvents, aromatic compounds, pesticides, and antibiotics [51]. *Micromonospora* exponentially grown cells featured a shift from odd-chain saturated fatty acids toward even ones (Table 1) as an adaptation mechanism and similarly to *Sphingomonas* K6 [52] and *Shewanella gelidimarina* [53,54] strains responding to the increase in the growth temperature. This fatty acid shift depends on the fatty acid synthetase specificity for primer molecules utilized to synthesize even fatty acids (acetyl-CoA) and odd ones (propionyl-CoA) in response to the growth temperature [53,55]. Such a cell response might derive from TeO_3_^2−^ and other heavy metal stress [56]. Additionally, *Micromonospora* cells highlighted a slight increase in the percentage of saturated fatty acids (i.e., C_18:0_ and *iso*-C_16:0_), decreasing the monounsaturated C_17:1_ (Table 1). This cell response correlates to the higher susceptibility of unsaturated fatty acids to free radicals [57] than saturated ones. Therefore, the latter can limit oxidative damage events determined by TeO_3_^2−^, whose toxicity enhances in the case of bacterial strains growing under aerobic conditions [58]. Moreover, the increase in the saturation degree of fatty acids is a common bacterial cell response to modulate membrane fluidity. This aspect relies on the higher phase transition temperature (T_M_) of saturated- and *iso*-branched fatty acids than unsaturated- and *anteiso*-branched ones [59]. Gram-positive strains feature a high proportion of *iso*- and *anteiso*-branched fatty acids, modulating their content in the cell membrane while responding to increased growth temperature or the presence of organic solvents, which tend to fluidize biological membranes [60,61,62]. Thus, the increased saturated- and *iso*-branched fatty acids in *Micromonospora* might rigidify the cell membrane in response to a high concentration (5 mM) of TeO_3_^2−^. The biological significance behind such microbial behavior likely concerns a structural adaptation of the bacterial membrane that opposes an excessive oxyanion entry into the cell cytosol, avoiding an oxidative burst determined by the pro-oxidant TeO_3_^2−^. A similar adaptation mechanism also occurs in the case of *Pseudomonas putida*, two strains of *Enterobacter intermedius*, and *Klebsiella pneumoniae* facing different cations (i.e., cadmium, nickel, copper, and zinc) [56].

### 3.2. Toxicity, Adaptation, and Recovery of Micromonospora Cells Growing in the Presence of Tellurite

A deeper understanding of the adaptation, resistance, and recovery of the *Micromonospora* strain facing TeO_3_^2−^ was obtained by evaluating the cell response in a time-course fashion.

#### 3.2.1. TeO_3_^2−^ Targets *Micromonospora* Cell Membrane

The prolonged lag phase observed in *Micromonospora* growth profiles (Figure 3b,c) is consistent with tellurite targeting the bacterial cell membrane (Figure 4b,c), compromising and impairing cell-reproductive growth and metabolic activity [58]. *Micromonospora* cells overcame this initial stress by undergoing morphological changes to either cope with or attenuate the external presence of tellurite. The bacterial hyphae appeared swollen and tightly packed (Figure 5), leading to the formation of floccules. Such evidence might result from changes occurring at the cell membrane and cell wall level, as reported for manganese-oxidizing or phototrophic bacteria facing chromate (CrO_4_^2−^), selenate (SeO_4_^2−^), or arsenate (AsO_4_^−2^) [63,64,65,66], and *Rhodococcus erythropolis* cells adapted to extreme conditions (i.e., high concentrations of either sodium chloride or copper sulfate) [67]. These morphology changes correlate with a protective mechanism devoted to finely regulating the attachment of oxyanions to the bacterial surface [68]. Moreover, *Micromonospora* cells secreted exudates resembling extracellular polymeric substance (EPS) in response to oxyanions, whose production increased as a function of TeO_3_^2−^ supplied (Figure 5). This evidence is in line with the drastic increase (*p* < 0.001) of integrals referring to -C-(OH) stretching at ca. 1310 cm^−1^ (Figure S9d) [69], corroborating EPS secretion noticed by SEM (Figure 5). Indeed, EPS represents the first-cell response to external stresses since it can bind toxic metal(loid) ions acting as a biosorbent [70,71,72], preventing their internalization and causing their external chelation. These events, in turn, allow bacteria to lower metal(loid) local concentration and, hence, their toxicity [69]. Furthermore, shifts of several polysaccharide vibrational modes (Tables S5–S7)—mainly deriving from peptidoglycan, teichoic, lipoteichoic, and teichuronic acids of the cell wall [27]—of bacterial cells incubated with TeO_3_^2−^ might indicate (i) the interaction of these macromolecules with the oxyanion, (ii) the modification of the produced EPS to increase its metal(loid)-binding ability, or (iii) the alteration of the peptidoglycan structure [22,69,73,74] to counteract the oxyanion stress. Similar observations applied to the variation of normalized integrals ascribed to IR polysaccharide contributions (Figure S9a–c). Specifically, the decrease in integrals referring to -CO stretching vibration (1040–1060 cm^−1^) observed for 100 μM TeO_3_^2−^_24h_, 250 μM TeO_3_^2−^_72h_, and 250 μM TeO_3_^2−^_120h_ (Figure S9b,c) might relate to the metalloid binding with polysaccharides [75]. The importance of polysaccharide variations also arose from PCA analysis, in which the vector describing these contributions distinguished unchallenged cells from those challenged (Figure 7). Interestingly, Goff and coworkers (2021) recently reported that sulfhydryl groups of the proteinaceous material within EPS of *Bacillus subtilis* play a crucial role in binding TeO_3_^2−^, mediating its sorption on bacterial surfaces [76]. At this stage, it cannot be ruled out that sulfhydryl groups in the EPS of *Micromonospora* EPS could bind TeO_3_^2−^ to limit its translocation into the cell cytoplasm.

TeO_3_^2−^ stress also caused a decrease in lipid production by *Micromonospora* growing cells, as highlighted by the drop of normalized integrals referring to asymmetric -CH_3_ stretching vibration (Figure S2a). This outline agrees with reports regarding the toxicity of several metals on either Gram-positive or Gram-negative bacterial strains [22,75,77]. Although the reason behind this cell response is still unclear, it may relate to the damage that TeO_3_^2−^ can trigger to the pyruvate dehydrogenase multienzyme complex responsible for the irreversible decarboxylation of pyruvate to acetyl-CoA, as described in the case of *Aeromonas caviae* ST [78]. This damage can affect both ATP synthesis [78] and lipid production, as acetyl-CoA is one of the precursors for fatty acid synthesis. For instance, the exposure of *Lipomyces starkeyi* to Cd^2+^ inhibited the acetyl-CoA formation determining a reduction in lipid generation and accumulation [79]. Moreover, the potentially lower amount of acetyl-CoA available to *Micromonospora* cells grown in the presence of this oxyanion will be reasonably devoted to re-generate energy at the expense of lipid biosynthesis. Additionally, the variations of both peak position and area of lipid -CH_x_ stretching vibrations in *Micromonospora* cells under TeO_3_^2−^ stress (Table S1; Figure S2) suggested the involvement of these macromolecules in the cell response to the oxyanion toxicity. TeO_3_^2−^ presence caused modifications in the cytosolic membrane’s permeability to protons and fluidity [73,77,80], as noticed for *Micromonospora* exponentially grown cells exposed to a very high TeO_3_^2−^ concentration (Table 2). A first fluidification effect was observed at the early stage of *Micromonospora* growth (24-h) under the TeO_3_^2−^ challenge (Figure 2, Figures S3 and S6d). This modification may derive from the oxidative stress exerted by TeO_3_^2−^ on bacterial cells, which can lead to the peroxidation of unsaturated fatty acids, decreasing membrane fluidity [69]. Similarly, cells grown for 72-h in the presence of 250 μM TeO_3_^2−^ featured a more fluid membrane than those incubated with 100 μM TeO_3_^2−^ (Figure S3), likely due to the higher toxicity exerted by the former, as confirmed by the observed prolonged lag-phase of growth (Figure 1c). At the late stages of *Micromonospora* growth in the presence of the oxyanion (72–120-h or 120-h for 100 μM or 250 μM TeO_3_^2−^, respectively), the lipid order in the membrane increased by decreasing the flexibility of the lipid acyl chain [73], as indicated by the shift towards bigger wavenumbers of the asymmetric -CH_2_ stretching vibration detected in ATR-FTIR spectra (Table S1). The same samples showed also high ν_as_ (CH_2_) normalized integrals (Figure S2b) that can relate to the introduction of saturation in fatty acids, or the elongation of their lipid acyl chains [80]. The former hypothesis was further corroborated by the increased A_νas(CH3)_/A_νas(CH2)_, A_νas(CH2)_/A_νs(CH2)_, and A_amide II_/A_νas(CH3)_ ratios, which are typical indicators of the fatty acid saturation degree [20,21,22,69,75], calculated for cells grown for 72 or 120-h in the presence of TeO_3_^2−^ rather than unchallenged cells (Figures S3 and S6d). These results are consistent with reports by Kepenek and coworkers for *Gordonia*, *Brevundimonas*, and *Microbacterium oxydans* exposed to metal(loids) [69]. Moreover, distinctive ATR-FTIR contributions of membrane modification were preponderant in the distribution of 100 μM TeO_3_^2−^_120h_ and 250 μM TeO_3_^2−^_120h_ within the 3D space identified by the three main PCs (Figure 5). Based on these observations, *Micromonospora* cells rigidified their cytoplasmic membrane during the late stages of growth to delay TeO_3_^2−^ entry into the cytoplasm, preventing ROS production, the generation of peroxidative products, and, in turn, membrane fluidification.

Another structural feature that emerged from SEM observations of *Micromonospora* cells facing oxyanion stress was the rising of membrane vesicle-like structures (MVs) (Figure 5f,j), whose study was mostly focused on Gram-negative bacteria, as these vesicles originate from the outer cell membrane, while Gram-positive ones feature a thick cell wall [81]. However, the study of such structures is lately gaining momentum also in the case of Gram-positive bacteria belonging to the Actinobacteria and Firmicutes phyla, where these MVs appeared to be heterogeneous in size [82], similarly to what was observed in this study. Interestingly, *Streptomyces coelicolor* M110 produced exudates—referred to as blue droplets featuring the antibiotic actinorhodin—that contained MVs carrying different proteins, which are associated with diverse cell functions, including energy metabolism, redox balance, and defense against oxidant agents. Indeed, such MVs were reported to contain catalase, TerB, and TerD proteins, which are involved in bacterial resistance toward tellurite [83]. Thus, it is reasonable to put forward the idea that a closely related strain, yet less investigated, such as *Micromonospora* might exploit vesiculation phenomena to face tellurite toxicity, although this aspect needs more dedicated research to obtain conclusive data.

#### 3.2.2. Involvement of Thiol-Containing Molecules

Similar to *Micromonospora* exponentially grown cells, TeO_3_^2−^ bioprocessing by those growing involved RSH-containing molecules, as indicated by the low amount of RSHs per given time (Figure 3d). However, a direct comparison between the profiles describing RSH depletion in *Micromonospora* growing cells with the available literature is challenging, as most studies focused on the earliest stages (up to 2.5-h) of either bacterial growth or exposure to this oxyanion. In the case of *Micromonospora* growing cells, RSH depletion may rely on (i) RSH groups within EPS binding with TeO_3_^2−^ [76], (ii) Painter-type reactions, (iii) ROS detoxification [84], and (iv) the modification of RSH-rich proteins caused by oxidative stress [27]. Hypotheses (i–iii) were sustained by the detection of vibrational modes attributable to RSH-, thiolate (RS^−^)-, and disulfide (RSSR)-containing molecules (Figure 6b,c; Table S1). Indeed, the binding of TeO_3_^2−^ with RSH groups of EPS proteins can lead to the formation of RS^−^ [76], Painter-type reaction involves all three RSH-deriving moieties, and RSSR groups can likewise derive from the transformation of ROS by low molecular weight RSHs [85]. The alteration of RSH-rich proteins consequently to TeO_3_^2−^ oxidative stress was suggested by the presence of IR contributions imputed to sulfinate (RSO_2_^−^), sulfinic (RSO_2_H), sulfonate (RSO_3_^−^), sulfonic (RSO_3_H), disulfide monoxide (RSOSR), and disulfide dioxide (RSO_2_SR) moieties (Table S1). Indeed, RSH-rich proteins interacting with ROS such as hydrogen peroxide (H_2_O_2_) can undergo the formation of RSSR bridges, S-thiolation (i.e., attachment of low molecular thiols), or cysteine overoxidation into RSO_2_H or RSO_3_H, even determining the loss of protein functions [27]. Protein modifications introducing RSOSR or ROS_2_SR moieties can instead arise from lipid peroxides, which, in turn, are generated from the peroxidation of polyunsaturated fatty acids by **^·^**OH [27].

#### 3.2.3. Peroxidation Products Deriving from Oxidative Stress

Variations in IR absorbance in the 1430–1000 cm^−1^ region (Figures S2, S7 and S8; Tables S1, S6 and S7) for *Micromonospora* cells incubated with TeO_3_^2−^ may relate to a decreased concentration of physiological macromolecules in favor of the peroxide counterparts, modifications in the structure and fluidity of the membranes, and the formation of α,β-unsaturated aldehydes during the breakdown of hydroperoxides or lipid peroxides [23,24]. Specifically, the high normalized -COO^−^ stretching integral of 100 μM TeO_3_^2−^_24h_ can be traced back to the partial bioprocessing of TeO_3_^2−^ at the early stage of growth, in line with the fluidification effect observed on the cell membrane (Figure S3). The similar amount of peroxidation products between TeO_3_^2−^-challenged and -unchallenged cells at 72-h of growth (Figure S8a) may relate to the increased production of EPS and membrane rigidification, which likely caused a slowdown in the oxyanion uptake. Although at 120-h of growth. cells analogously produced EPS and rigidified their membrane (Figures S3 and S6d), the continuous bioprocessing of TeO_3_^2−^ for 48-h more might have triggered the generation of a large amount of ROS, which can exasperate the generation of cellular peroxides (Figure S8a). This phenomenon seemed more relevant for 250 μM TeO_3_^2−^_120h_, as suggested by its importance in discriminating the localization of this sample in the 3D space of PCA (Figure 7). In line with these observations, the production of peroxidation products, either as peroxides lipids or oxidized (carbonylated) proteins, was previously reported for *Escherichia coli* strains facing TeO_3_^2−^ [46,86,87,88].

#### 3.2.4. Protein Aggregation and Phosphorylation

In addition to protein oxidation, ROS stress can also cause variations in the secondary structure of proteins. Indeed, Kiwi and coworkers related peroxidation phenomena to the appearance of three Amide I peaks attributable to α-helix (ca. 1655 cm^−1^), β-antiparallel (ca. 1687 cm^−1^), and β-sheet (ca. 1636 cm^−1^) structures, and two Amide II vibrations centered at 1545 and 1517 cm^−1^ [23]. These results agree with those observed for *Micromonospora* cells at different growth stages in the presence of TeO_3_^2−^ (Figures S5 and S6). Particularly, hydrophobic β-sheet structures are involved in protein aggregation, which is related to cell growth rates as well as external stressors [89]. Indeed, bacterial cells entering the stationary phase feature protein aggregates due to the low ATP availability, which regulates chaperones’ functionality and protein solubility in the intracellular environment [89]. This observation is in line with the build-up of β-sheet secondary structures observed for *Micromonospora* unchallenged cells after 72-h of growth (transition phase) (Figure 3a and Figure S5a). Moreover, upon cell division, protein aggregates are distributed to the progeny at each cell cycle [89]. This phenomenon may also occur in filamentous bacteria, as an elevated amount of β-sheet structures was detected for *Micromonospora* unchallenged cells in their RG2 phase (Figure 3a and Figure S5a). ROS and oxyanions (i.e., arsenite) can amplify or even cause protein aggregation through covalent modification of amino acid side chains, damage and inhibition of chaperones, and ATP depletion [89,90]. Hence, the increase in β-sheet-containing proteins at the early stage (24-h) of *Micromonospora* growth in the presence of TeO_3_^2−^ (Figure S5b,c) may derive from the oxyanion per se and its pro-oxidant effect. For the sake of argument, modifications in the protein secondary structure (i.e., β-sheet motifs) were also observed in Gram-positive microorganisms exposed to metal cations [75,91]. However, protein aggregation can regulate gene expression and sequester proteins involved in cellular processes vulnerable to external stress [89]. The conditional aggregation of such proteins allows bacteria to reallocate cellular resources to promote cell growth, protect cell integrity and function, and cope with external stresses threatening the cell proteome [89]. This aspect might partially explain the recovery of *Micromonospora* TeO_3_^2−^-challenged cells from 72-h of growth onwards (Figure 3b,c).

Protein phosphorylation can also assist the recovery process of bacterial cells facing external stresses. In actinomycetes, phosphorylated proteins can regulate gene expression and signaling, protein biosynthesis, central metabolism, membrane transport, and cell division [92]. For instance, in *S. coelicolor* A3(2), the protein folding relies on the phosphorylation of the chaperonin GroES enabling its interaction with GroEL [92], whereas *S. reticuli* features a redox sensing system in which phosphorylation reactions allow bacterial cells to respond and adapt to pro-oxidant agents [93]. Moreover, protein phosphorylation plays a role in the oxidative stress handling of *S. toyocaensis* NRRL 15009, as the disruption of the gene coding for a membrane-bound kinase causes the loss of specific responses to ROS-generating compounds [94]. These examples highlight how oxidative stress response management is crucial in soil-dwelling actinomycetes such as PSB *Micromonospora* spp. and can justify protein phosphorylation levels observed for *Micromonospora* cells growing in the presence of TeO_3_^2−^ (Figure S8b). The high degree of phosphorylation detected in up to 120-h of growth in cells challenged with 250 μM TeO_3_^2−^ may indicate that this post-translational modification can trigger mechanisms devoted to cell recovery (Figure 3c) from the oxyanion stress. In line with this hypothesis, 100 μM TeO_3_^2−^ caused increased protein phosphorylation only when cells suffered the oxyanion toxicity the most (lag phase; Figure 3b and Figure S8b), suggesting a concentration-dependent cell response to TeO_3_^2−^. The relevance of protein aggregation and phosphorylation as responses elicited by *Micromonospora* cells experiencing TeO_3_^2−^ toxicity was further confirmed by the clustering of 100 μM TeO_3_^2−^_24h_ and 250 μM TeO_3_^2−^_72h_ samples in the region of the 3D space described by vectors referring to β-strand and random coil secondary structures and protein phosphorylation (Figure 7). In addition to protein phosphorylation, another mechanism of oxidative stress recovery for *Micromonospora* cells facing TeO_3_^2−^ was the induction of SOD enzymes, in line with previous reports [41,45,46]. Similar to protein phosphorylation, SOD induction depended on the oxyanion concentration supplied (Figure 3e). This aspect is likely to be ascribed to the oxidation products (i.e., superoxide anions)—which need to be dismantled to achieve a full cell recovery—derived from the transformation of TeO_3_^2−^ mediated by RSHs. Despite the above-mentioned strategies elicited by the *Micromonospora* strain to handle this oxyanion, bacterial cells only removed ca. 130 μM TeO_3_^2−^ (Figure 3b,c). This outline suggests the existence of a TeO_3_^2−^ threshold concentration that this strain can handle, which likely links to the saturation of biochemical assets required to remove and process oxyanions [95] and attenuate the derived oxidative damage.

## 4. Materials and Methods

### 4.1. Bacterial Strain, Growth Medium, and Culture Conditions

The *Micromonospora* strain was pre-cultivated in 250 mL Erlenmeyer Baffled Flask containing 50 mL of the R5 medium (composed (g L^−1^)of potassium sulfate (K_2_SO_4_; 0.25), magnesium chloride hexahydrate (MgCl_2_·6H_2_O; 10.17), glucose (10), casaminoacids (0.1), yeast extract (5), and 3-(N-morpholino)propanesulfonic acid sodium salt (MOPS; 21)) amended with (2 mL per liter of medium) a solution of trace elements (composed (mg L^−1^) of zinc chloride (ZnCl_2_; 40), iron (III) chloride hexahydrate (FeCl_3_·6H_2_O; 200), copper (II) chloride dihydrate (CuCl_2_·2H_2_O; 10), manganese (II) chloride tetrahydrate (MnCl_2_·4H_2_O; 10), sodium tetraborate decahydrate (Na_2_B_4_O_7_·10H_2_O; 10), and ammonium molybdate tetrahydrate ((NH_4_)_6_Mo_7_O_24_·4H_2_O; 10)) for 8 days at 30 °C with shaking (180 rpm) to synchronize bacterial cells through nutrient starvation. Afterward, bacterial cells were inoculated (2% *v*/*v*) in a fresh R5 medium (50 mL) and grown for 168-h (8 days) in the presence of different potassium tellurite (K_2_TeO_3_) concentrations (i.e., 100, 250, and 500 μM). The bacterial growth profile was evaluated by quantifying the total protein content isolated from bacterial culture aliquots (1 mL) collected every 24-h. Data are expressed as protein content (mg mL^−1^) with standard deviations (SD; *n* = 5). Concomitantly, every 24-h—up to 168-h—aliquots (500 μL) of bacterial cultures were sampled and centrifuged 10,000× *g* for 15 min to recover the cell-free spent medium and the corresponding biomass pellets to perform tellurite consumption and thiol oxidation assays, respectively.

Exponentially grown *Micromonospora* cells were obtained by harvesting biomasses at their RG2 phase reached in the R5 medium. Bacterial biomass was then washed twice with 50 mL of phosphate buffer saline (PBS; composed (g L^−1^) of sodium chloride (NaCl; 8), potassium chloride (KCl; 0.2), disodium hydrogen phosphate (Na_2_HPO_4_; 1.44), and potassium dihydrogen phosphate (KH_2_PO_4_; 0.24)) pH 7.4 through centrifugation steps performed at 6000× *g* for 15 min to remove any residual R5 medium. After the washing steps, cells were resuspended in PBS and amended with 100 μM K_2_TeO_3_. Exponentially grown biomass and the corresponding cell-free spent medium was collected, as described above, every hour—up to 6-h—to perform TeO_3_^2−^ uptake and thiol oxidation assays.

All the reagents were purchased from Merck Life Science S.r.l. (Milan, Italy).

### 4.2. Tellurite Consumption and Uptake Assays

The capability of the *Micromonospora* strain to remove TeO_3_^2−^ over the considered timeframe was evaluated as published elsewhere [96]. Briefly, 10–100 μL aliquot of cell-free spent medium was mixed with 600 μL of 0.5 M Tris-HCl buffer pH 7.0, 200 μL of diethyldithiocarbamate, and R5 medium up to 1 mL volume. The absorbance of the above-described mixture was read at 340 nm through an Eppendorf D30 BioPhotometer^®^. The residual TeO_3_^2−^ concentration (μM) in the spent medium was determined by fitting the absorbance value to a calibration curve, which derives from the spectrophotometric analysis of solutions containing a known concentration (10, 50, 100, 250, and 500 μM) of TeO_3_^2−^ (R^2^ = 0.9846). Data are reported as average values of biological replicates (*n* = 5) with SD.

As for TeO_3_^2−^ uptake experiments, the cell-free spent medium collected from exponentially grown *Micromonospora* cells was processed as above-described and assayed to estimate the residual TeO_3_^2−^ content either in the absence or presence of 50 μM of the potent protonophore and electron-transfer uncoupler CCCP. Data are expressed as average values (*n* = 3) of TeO_3_^2−^ nmols taken up by *Micromonospora* cells normalized for the total protein content (grams) estimated for each time-point considered with SD.

All the reagents were purchased from Merck Life Science S.r.l. (Milan, Italy).

### 4.3. Thiol Oxidation Assay

The oxidation of the RSHs because of the challenge exerted by TeO_3_^2−^ was evaluated for the *Micromonospora* strain either as exponentially grown or growing cells as reported elsewhere [40]. Briefly, cells were resuspended in 1 mL of a solution containing 50 mM of Tris-HCl pH 8.0, 5 mM of ethylenediaminetetraacetic acid (EDTA), 0.1% *v/v* of sodium dodecyl sulfate (SDS), and 0.1 mM of 5,5′-dithiobis(2-nitrobenzoic acid) (DTNB) and incubated at 37 °C for 30 min. After this step, all samples were centrifuged at 15,000× *g* for 10 min, the absorbance of supernatants being read at 412 nm (SPECTROstar^®^ Nano, BMG Labtech, Milan, Italy) using 1-cm path-length glass cuvettes. Considering the DTNB molar extinction coefficient (13,600 M^−1^ cm^−1^) at this wavelength (412 nm), this value was used to calculate RSH concentrations isolated from cell samples. Similarly, samples were processed at each time point to estimate the protein content to normalize RSH concentration values (RSH μmols g protein^−1^). The amount of RSH calculated at time 0 was then subtracted from those evaluated over the timeframe of the assay, therefore, reporting the data as the average value (*n* = 3) of the loss of reduced RSHs from the original pool with SD.

All the reagents were purchased from Merck Life Science S.r.l. (Milan, Italy).

### 4.4. Cell Viability Assay

*Micromonospora* cells growing for 24-h either in the absence or presence of 100 or 250 μM TeO_3_^2−^ were collected and washed three times with PBS and then labeled with the Live/Dead^®^ BacLight^™^ (Molecular Probes, Merck Life Science S.r.l. Milan, Italy) stain for 30 min at room temperature, as published elsewhere [97]. This kit provides two fluorescent dyes, namely: SYTO 9 can emit green fluorescence and labels all bacterial cells in a population, while propidium iodide fluoresces in the red region of the visible spectrum, entering only bacterial cells with damaged membranes. Thus, each labeled bacterial sample was imaged through a Leica DM2500 fluorescence microscope with a 20× objective. Micrographs were captured using red and green fluorescence filters and merged by ImageJ software 1.53a (Bethesda, MD, USA).

### 4.5. Assessment of the Membrane Potential

Exponentially grown *Micromonospora* cells were challenged for 6-h at 30 °C with shaking (180 rpm) with 100 μM TeO_3_^2−^. CCCP (50 μM) was used as a positive control, while unchallenged bacterial cells were used as a reference. After the challenge, bacterial cells were washed three times and resuspended with a permeabilization buffer (PB; composed of 10 mM Tris-HCl pH 7.4, 1 mM EDTA, and 10 mM glucose) amended with the membrane potential cationic dye JC-1 (10 μg mL^−1^; Invitrogen^™^, Waltham, MA, USA), and labeled for 30 min at 30 °C with shaking (180 rpm). Bacterial cells loaded with the JC-1 dye were then washed three times with PB to remove the dye in excess, then aliquoted (150 μL) into a multitier 96-well plate. The JC-1 fluorescence emission shift from the red (590 nm) to the green (530 nm) region of the visible spectrum was collected using a plate reader (Synergy HT Biotek, Winooski, VT, USA) upon its excitation at 488 nm. Concomitantly, cell samples (150 μL) were utilized to isolate the total protein content to normalize the red-to-green (590 nm/530 nm) fluorescence ratio. Additionally, the basal fluorescence emission of JC-1 in PB only was subtracted from the obtained fluorescence ratios. The data are reported as average values (*n* = 3) of JC-1_590 nm/530 nm_ (Intensity Counts g protein^−1^) with SD. Each bacterial sample was also imaged through fluorescence microscopy, and the images were processed as described above (Section 4.4).

### 4.6. Fatty Acid Methyl Esters Analysis

The whole-cell fatty acids were isolated from *Micromonospora* exponentially grown cells after their exposure to a high TeO_3_^2−^ concentration (5 mM) for 6-h, as reported elsewhere [98]. Bacterial cells were centrifuged for 15 min at 6000× *g*, washed three times to remove any trace of the culture broth, homogenized to avoid cell floccules, and resuspended in 15 mL of 0.2 M of potassium hydroxide (KOH) in methanol and 0.1 mg mL^−1^ of the internal standard nonadecanoic acid (C_19:0_). Bacterial cells were incubated at 37 °C for 1-h and vortexed every 10 min, allowing for the release and methylation of fatty acids. Afterward, the samples were amended with 3 mL of acetic acid (1 M) and 10 mL of hexane to extract fatty acid methyl esters (FAMEs). The hexane phase was dried up by a rotavapor, then samples were resuspended in 200 μL of hexane and subsequently analyzed using a Thermo Scientific FOCUS^™^ gas chromatography equipped with a flame ionization detector (FID) and a fused-silica capillary column Mega-10 having the following characteristics: 50 m × 0.32 mm I.D.; film thickness 0.25 μm. The utilized thermal program featured an initial isotherm at 115 °C for 5 min followed by a temperature increase up to 230 °C at a rate of 1.5 °C per minute, and a final isotherm was carried out for 2 min at 230 °C. FAME peaks were identified relying on the retention times of known fatty acid standards (Supelco bacterial acid methyl esters and Supelco 37 Component FAME). The data were expressed as relative percentage abundance of each fatty acid with SD (*n* = 3) with respect to total fatty acids.

### 4.7. ROS Determination

The exponentially grown cells were incubated with 100 μM TeO_3_^2−^ as described in Section 4.5. Afterward, the bacterial cells were washed three times with PBS and incubated—for 1-h at 30 °C (180 rpm)—with 50 μM of 2′,7′-dichlorofluorescein diacetate (DCF). Cells were then washed three times with PBS and resuspended in fresh PBS buffer, being samples (150 μL) aliquoted in a multitier 96-well plate. At the same time, aliquots (150 μL) of bacterial cells were processed to estimate the total protein content to normalize DCF fluorescence emission, which was collected at 525 nm upon its excitation at 488 nm (Synergy HT Biotek). Furthermore, the basal fluorescence emission of DCF in PBS only was subtracted from the obtained fluorescent signals deriving from bacterial cells. Unchallenged cells were used to compare the physiological level of ROS produced during the assay’s considered timeframe (0–6-h). The data are reported as average values (*n* = 3) of DCF fluorescence emission (Intensity Counts g protein^−1^) with SD.

All the reagents were purchased from Merck Life Science S.r.l. (Milan, Italy).

### 4.8. SOD Activity Evaluation

*Micromonospora* cells growing for 24, 72, and 120-h in the absence/presence of TeO_3_^2−^ (i.e., 100 and 250 μM) were sampled and processed to isolate the soluble protein fraction. Briefly, bacterial cell pellets were washed with and resuspended in an extraction buffer (EB) containing a cocktail of protease and phosphatase inhibitors such as phenylmethylsulfonyl fluoride (PMSF; 0.5 mM), leupeptin (5 μg mL^−1^), benzamidine (4 μg mL^−1^), pepstatin (7 μg mL^−1^), Tris-HCl (10 mM) pH 7.4, and EDTA (5 mM). Afterward, cells were lysed through 5 steps (30 s each interspersed by 30 s of pause on ice) of sonication performed at 15 W (Vibra-Cell^™^ Sonics and Materials Inc. Danbury, CT, USA). Proteins were separated from cell debris through a centrifugation step performed at 8000× *g* for 15 min at 4 °C and precipitated by adding ice-cold acetone (2 volumes) for 1-h at −80 °C. Samples were then centrifuged at 15,000× *g* for 20 min at 4 °C, the supernatant removed, while protein pellets were dried by speed vacuum (Eppendorf^®^ 5301 concentrator; Milan, Italy). Finally, protein samples were resuspended in EB and quantified by the Bradford assay. The SOD activity test was performed using 5 μg of the soluble protein fraction and according to the manufacturer protocol of the superoxide dismutase activity kit (Sigma-Aldrich^®^, Milan, Italy). The data are reported as the average value (*n* = 3) of the SOD activity percentage (%) with SD.

All the reagents were purchased from Merck Life Science S.r.l. (Milan, Italy).

### 4.9. Scanning Electron Microscopy (SEM) Imaging

The *Micromonospora* strain was cultivated in the R5 medium either in the absence or presence of two different TeO_3_^2−^ concentrations (i.e., 100 and 250 μM) for 24, 72, and 120-h of growth. Afterward, cells were pelleted at 8000× g for 10 min, washed twice with sterile saline (0.9% *w*/*v*) solution, and resuspended in a 2.5% (*v*/*v*) glutaraldehyde solution. The samples were fixed overnight (ca. 18-h) at 4 °C. The day after, cells were pelleted as described above and dehydrated through three washing steps (10 min each) with increasing concentrations (30, 40, 50, 60, 70, 80, 90% *v*/*v*, and absolute) of ice-cold ethanol. Finally, cells were deposited onto carbon-coated copper grids (300 mesh), sputter coated with gold (Sputtering Scancoat Six, Edwards) for 60 s under an argon atmosphere, and observed using a FEG-SEM microscope (QUANTA 200F, FEI) with an accelerating voltage of 10 kV, as previously described [99].

### 4.10. ATR-FTIR Spectroscopy

ATR-FTIR spectroscopy was performed on bacterial cells grown for 24, 72, and 120-h either in the absence (Reference) or in the presence of 100 and 250 μM TeO_3_^2−^ by using a μFTIR Lumos (Bruker, UK) equipped with a Platinum ATR and IR microscope featuring 0.1 μm as lateral resolution. The spectra were collected in triplicate (*n* = 3) in the 600–4000 cm^−1^ region (resolution of 2 cm^−1^), and 120 scans were registered per sampling point. The obtained spectra were analyzed through OPUS7.5 (Bruker Instruments, Billerica, MA, USA) and OriginPro 2016 software [27]. IR bands were assigned according to [100,101,102,103,104,105,106,107,108,109,110]. The peak integrals (area) of interest obtained through spectral deconvolutions were duly normalized to highlight differences deriving more precisely from the stress exerted on bacterial cells by TeO_3_^2−^. Indeed, peak integrals obtained for -CHx stretching vibrations or amide I bands were normalized against the integrals calculated in the 2960–2850 cm^−1^ or 1690–1600 cm^−1^ region of the spectra, respectively. Peak integrals referring to (i) the asymmetric -COO- stretching vibration of peroxidation products (1430–1360 cm^−1^), (ii) the -C-O-P stretching of phosphorylated proteins (ca. 1235 cm^−1^), and typical polysaccharides contributions (i.e., 1150, 1105, 1079, and 1040 cm^−1^) were normalized against the IR integrals of the 1750–950 cm^−1^ region.

### 4.11. Statistical Analysis

Statistical analysis of the obtained results was carried out through the Student’s *t*-test (OriginPro software package) to compare means between the samples’ datasets. The statistical significance of the observed differences between datasets was considered when *p* < 0.05.

Multivariate statistical analysis was performed on normalized IR integrals to evaluate macromolecules involved in TeO_3_^2−^ bioprocessing and their modifications determined by the oxyanion toxicity. PCA was chosen as a multivariate statistical analysis and was performed using the dedicated package of OriginPro 2016 software as described elsewhere [27]. PCA was carried out by constructing a correlation matrix in which the observations were samples in triplicate (*n* = 27) analyzed through ATR-FTIR spectroscopy and the variables were normalized integrals referring to (i) polysaccharides’ presence (1200–950 cm^−1^), (ii) peroxidation products (1430–1360 cm^−1^), (iii) α-helix structures (1660–1650 cm^−1^), (iv) β-strand (1690–1660 and 1640–1610 cm^−1^) and random coil (1650–1640 cm^−1^) structures, (v) additional amide II bands (1560–1550, 1535–1520 cm^−1^), and integral ratios obtained for (vi) protein phosphorylation (A_ν (C-O-P)_/A_amide_) and (vii) membrane modification (A_νas (CH2)_/A_νas(CH3)_, A_νas (CH2)_/A_νs(CH2)_, and A_amide II_/A_νas(CH3_).

## 5. Conclusions

The present study combines biological, physical-chemical, and statistical approaches to deeply characterize a non-pathogenic and soil-dwelling *Micromonospora* strain facing TeO_3_^2−^ toxicity. Exploiting different bacterial cell physiologies allowed us to delineate the mechanism(s) elicited by the *Micromonospora* strain to tolerate, adapt, and respond to the presence of this oxyanion.

TeO_3_^2−^ immediate toxicity was evaluated on *Micromonospora* exponentially grown cells exposed to two oxyanion concentrations highlighting how these chemical species (i) primarily target the bacterial cell membrane, (ii) enter the intracellular milieu likely through a ΔpH-dependent transporter, and (iii) are processed through RSH-containing molecules, giving rise to oxidative damage. Nevertheless, when challenged with a very high TeO_3_^2−^ concentration (5 mM), exponentially grown cells coped with the oxyanion by inducing membrane modifications most likely involved in controlling TeO_3_^2−^ homeostasis. Similar results were obtained for *Micromonospora* cells growing in the presence of this oxyanion, yet they recovered from this stress over time. The cell recovery seems to involve EPS production, cell membrane rigidification, protein phosphorylation, SOD induction, and, to some extent, protein aggregation. The oxyanion toxic effects and the adaptation mechanisms behind the cell recovery depended on the TeO_3_^2−^ concentration supplied. Indeed, *Micromonospora* cells facing 250 μM TeO_3_^2−^ featured emphasized cell responses, as highlighted by a prolonged lag phase and the lack of the RG2 growth phase typical of unchallenged cells and those growing in the presence of 100 μM TeO_3_^2−^.

This study expands the knowledge about the metal(loid)-microbe interactions occurring in a poorly investigated bacterial genus revealing that TeO_3_^2−^ acts directly or indirectly on different cell targets. This outcome indicates that the mechanism(s) of both oxyanion toxicity and resistance of *Micromonospora* cells is highly complex and involves several players. Moreover, the latter must be considered and investigated to better unveil the potential biotechnological application of *Micromonospora* spp. in metal(loid) recovery.

## Figures and Tables

**Table 1:** The relative percentage of total fatty acids isolated from *Micromonospora* cells exposed for 6-h to TeO_3_^2−^.

	Relative Abundance (%)	
Fatty Acid	Control Cells	TeO_3_^2−^-Challenged Cells
C_15:0_	9.88 ± 0.26	3.67 ± 0.80
C_16:0_	4.59 ± 0.07	3.64 ± 0.01
C_17:0_	18.0 ± 0.2	10.3 ± 0.6
C_18:0_	3.56 ± 0.27	8.95 ± 0.97
*iso-*C_15:0_	6.23 ± 0.03	8.27 ± 0.25
*anteiso-*C_15:0_	3.09 ± 0.08	2.78 ± 0.01
*iso-*C_16:0_	12.4 ±0.9	22.6 ± 1.4
*iso-*C_17:0_	1.93 ± 0.09	3.37 ± 0.28
C_16:1ω7c_	3.02 ± 0.21	4.78 ± 0.11
C_17:1_	28.0 ± 0.9	20.2 ± 0.9
C_18:1ω9c_	9.09 ± 0.23	11.7 ± 1.7
Even saturated fatty acid	20.5 ± 1.3	36.1 ± 0.5
Odd saturated fatty acid	39.4 ± 1.2	28.2 ± 0.5
Saturated fatty acid	59.9 ± 0.8	64.3 ± 0.9
Unsaturated fatty acid	40.1 ± 0.5	35.7 ± 0.9
Saturated/Unsaturated	1.49 ± 0.04	1.80 ± 0.07

ω indicates the double bond position closest to the methyl end. *Iso* and *anteiso* indicate the position of the methyl-substituent at the penultimate and/or antepenultimate carbon of the carboxyalkyl chain.

**Table 2:** Parameters (Principal components and discriminating variable vectors) determined through PCA.

	PC1	PC2	PC3
**Information (%)**	44.0	28.0	17.4
**Discriminating ^1^ Vectors**	protein phosphorylation	amide II_II_	polysaccharides
	membrane integrity	polysaccharides	β-strand + random coil structure
	peroxidation products		α-helix structure
	polysaccharides		

^1^ Discriminating vectors are those that describe variables separating PCs.

## Data Availability

Not applicable.

## Supplementary Materials

The following supporting information can be downloaded at: https://www.mdpi.com/article/10.3390/ijms232012631/s1.
