# Supplementary material for: Tolerance, Adaptation, and Cell Response Elicited by Micromonospora sp. Facing Tellurite Toxicity: A Biological and Physical-Chemical Characterization
Source: Int J Mol Sci. 2022 Oct 20;23(20):12631. doi: 10.3390/ijms232012631 (PMC9604092; doi:10.3390/ijms232012631)
Supplement: Supplementary file 1 [file ijms-23-12631-s001.zip › ijms-1940622-supplementary.pdf]

International Journal of *Molecular Sciences*

MDPI

Supplementary Material for

Tolerance, adaptation, and cell response elicited by *Micromonospora* sp. facing tellurite toxicity: a biological and physical-chemical characterization

Elena Piacenza [1,*], Rosa Alduina [1], Simona Campora [1], Francesco Carfì Pavia [2], Delia Francesca Chillura Martino [1], Vito Armando Laudicina [3], Raymond Joseph Turner [4], Davide Zannoni [5], Alessandro Presentato [1,*]

[1] Department of Biological, Chemical and Pharmaceutical Science and Technologies, University of Palermo, Viale delle Scienze, Ed. 16, 90128, Palermo, Italy
[2] Department of Engineering, University of Palermo, Viale delle Scienze, Ed. 8, 90128, Palermo, Italy
[3] Department of Agricultural, Food and Forest Sciences University of Palermo, Viale delle Scienze, Ed. 4, 90128, Palermo, Italy
[4] Department of Biological Sciences, University of Calgary, 2500 University Drive NW, Calgary, AB T2N 1N4, Canada
[5] Department of Pharmacy and Biotechnology, University of Bologna, Via Irnerio 42, 40126, Bologna, Italy
* Correspondence: elena.piacenza@unipa.it (E.P.); alessandro.presentato@unipa.it (A.P.)

Citation: Lastname, F.; Lastname, F.; Lastname, F. Title. *Int. J. Mol. Sci.* **2022**, *23*, x. https://doi.org/10.3390/xxxxx

Academic Editor: Firstname Lastname

Received: date
Accepted: date
Published: date

Publisher's Note: MDPI stays neutral with regard to jurisdictional claims in published maps and institutional affiliations.

Copyright: © 2022 by the authors. Submitted for possible open access publication under the terms and conditions of the Creative Commons Attribution (CC BY) license (https://creativecommons.org/licenses/by/4.0/).

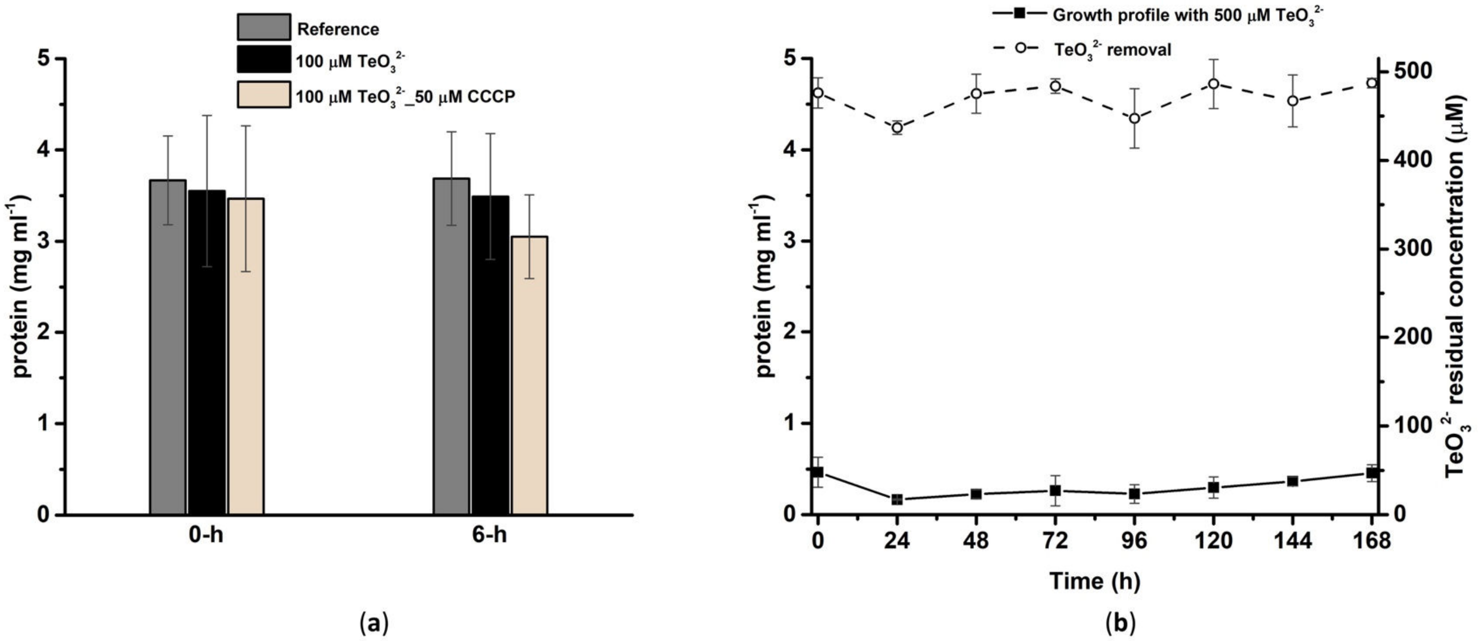

Figure S1. Evaluation of the protein content of *Micromonospora* exponentially grown cells (**a**) and growth profile and TeO_3^{2-} bioconversion of *Micromonospora* cells growing in the presence of 500 μM TeO_3^{2-}.

Table S1. ATR-FTIR absorption bands and identification of *Micromonospora* unchallenged cells or exposed to TeO_3^{2-}.

$\tilde{\nu}$ (cm^{-1})									Vibrational modes	Identification
**Reference$_{24h}$**	**Reference$_{72h}$**	**Reference$_{120h}$**	**100 μM $TeO_3^{2-}$$_{24h}$**	**100 μM $TeO_3^{2-}$$_{72h}$**	**100 μM $TeO_3^{2-}$$_{120h}$**	**250 μM $TeO_3^{2-}$$_{24h}$**	**250 μM $TeO_3^{2-}$$_{72h}$**	**250 μM $TeO_3^{2-}$$_{120h}$**		
	3431(s)								ν (OH)	Carbohydrates [1]
3279	3287	3286	3287	3287	3271	3297	3290	3290	ν (NH)	Proteins (Amide A) [2]
3069(s)	3065(s)	3068(s)	3073(s)	3063(s)	3063(s)	3061(s)	3075(s)	3062(s)	ν_s (NH^{3+})	Proteins (Amide B) [3]
2953	2953	2954	2957	2955	2957	2960	2960	2962	ν_{as} (CH_3)	Fatty acids [2–4]

2924	2922	2924	2922	2926	2922	2920	2926	2923	ν_{as} (CH_2)	Fatty acids [2–4]
2872	2873	2872	2870	2872	2873	2870(s)	2872	2871	ν_s (CH)	Amino acids in fatty acids [4]
2851	2852	2852	2855	2856	2855	2851	2855	2856	ν_s (CH_2)	Fatty acids [2–4]
1737	1740	1737	1735	1735	1740	1739	1742	1740	ν (CO)	Ester moieties of lipids and polysaccharides [2–4]
		1715(s)	1717	1715					ν (CO)	R-CO-CH_3 ketones [5]
					1686(s)				ν (CO); δ (NH)	β-antiparallel proteins (Amide I) [2–4]
1653 (s)	1657(s)		1654			1656(s)	1658(s)		ν (CO)	α-helix proteins (Amide I) [2–4]
1648	1649	1650	1646	1645	1646(s)	1650	1648(s)	1648(s)	ν (CO); δ (NH)	Random coil proteins (Amide I) [6]
1636	1642	1639	1638	1635	1638	1640	1641	1640	ν (CO); δ (NH)	β-sheet proteins (Amide I) [2]
						1633	1630(s)	1630	ν (CO); δ (NH)	β-sheet proteins (Amide I) [2]
1617(s)									ν (CO); δ (NH)	β-sheet proteins (Amide I) [2]
		1593							δ_{as} (NH_3^+)	Amino acid residues [3]
1577	1565	1562(s)	1577(s)			1563	1565(s)	1562(s)	ν_{as} (COO^-)	Polysaccharides [2]
1558(s)	1555(s)	1552(s)	1553(s)				1552(s)		δ (NH); ν (CN)	Amide II of proteins [2–4]
	1548				1548(s)	1547			δ (NH); ν (CN)	Amide II of proteins (Amide II) [2–4]
1541	1537	1539	1542	1542	1541		1540	1542	δ (NH); ν (CN)	Amide II of proteins (Amide II) [2–4]
			1534			1536		1535	δ (NH); ν (CN)	Amide II of proteins (Amide II) [2–4]
1522(s)	1520(s)		1522						δ_s (NH^{3+}); δ (NH); ν (CN)	Amino acid residues [3]; RSH-containing molecules [7]
1510		1509(s)	1509(s)	1510(s)	1510(s)	1515(s)		1511(s)	δ_s (NH_3^+)	Amino acid residues [3]; RSH-containing molecules [7]
1490		1496(s)	1495(s)	1494(s)	1495(s)		1499(s)	1501(s)	δ_s (NH_3^+)	Amino acid residues [5]
1473									δ_{sciss} (CH_2)	Lipids and proteins [4]
1464	1468	1469	1465	1467	1466(s)	1465(s)	1468	1467	δ (CH_2); δ (CH_3); β (CH_2)	Lipids and proteins [4]
1457			1457					1459	δ_{sciss} (CH_2); δ (OH); ν CC(O); ν_s (COO^-)	Polysaccharides [8]; aliphatic groups of proteins [9]; R-SO_2H-containing molecules [7]

1450	1454	1455	1448	1455	1451	1452	1453	1451	δ_{sciss} (CH_2); δ (OH); ν CC(O)	Polysaccharides [8]; aliphatic groups of proteins [9]; R-SO_2H-containing molecules [7]
1438	1443	1442	1438			1443(s)	1440(s)		δ_{sciss} (CH_2); δ (OH); ν CC(O); ν_s (COO^-)	Polysaccharides [8]; aliphatic groups of proteins [9]; R-SO_2H-containing molecules [7]
1418	1413(s)	1414	1419(s)						ν_s (COO^-)	Peroxidation products [5]
1397	1400	1401	1397	1400	1402	1402	1398	1399	ν_s (COO^-)	Amino acid side chains; free fatty acids [4]; peptides [9]
1384(s)	1384(s)	1385(s)	1380(s)	1382(s)	1379(s)	1382		1385	δ (CH); δ (OH); δ (COH); β (CH_3); ν CC(O); ν_s (COO^-); δ (NH_2); ν (CN)	Aldehydes, carboxylic acids, peptides [9]; aromatic amines [10]
	1365(s)	1364	1373(s)		1373(s)				β (CH_3); δ (CH); ν_s (COO^-)	Lipids and proteins [4]; RSH-, RS^--, RSO_2^--, RSO_3H-, RSSR-, RSOSR, and RSO_2SR-containing molecules [7]
	1345								β C(OH); δ (OH); δ (CH); ν_s (COO^-)	Polysaccharides [4,8]; RSH-, RS^--containing molecules [7]
1339	1335	1340	1340	1339	1340	1335		1340	β C(OH); δ (OH); δ (CH); ν_s (COO^-)	Polysaccharides [4,8]
1315	1313	1314	1314	1315	1312				ν (C-OH)	Polysaccharides in EPS [11]
					1304(s)	1306	1306	1305	ν (C-OH)	Acetic acid [5]
					1286(s)				ω (CH_2); ν (CN); δ (NH_2); $\varrho_{as(oph)}$ (CH); ν (COC); ν (CCO);	Ester moieties [2]; proteins [4]; Acetic acid [5]; RSH-, RSO_2^--, RSO_3^-, RSO_2H-, RSO_3H-, RSSR-, RSOSR-containing molecules [7]

									δ (OH); δ (CH); ν (C-OH)	
1239	1237	1248(s)	1239	1238	1241	1238	1240	1238	ν (CN); δ (NH_2)	Proteins (Amide III) [2]
		1235			1235(s)				β (NH); ν_{as} (PO_2^-)	Nucleic acids [4]
1218(s)									ν_s (PO_2^-)	Teichoic and lipoteichoic acids [7]
		1202							ν_s (PO_2^-)	Teichoic and lipoteichoic acids [7]
1171(s)			1170(s)				1173		ν ($C{=}O$)$_{ring}$	Polysaccharides [5]
1150	1149	1150	1154	1151	1148	1154	1148	1152	ν_{as} (COC); δ (CH_2); δ (CH); δ (NH_2); ν (CO) δ (OH); δ (S)OH	Nucleic acids; $\alpha_{(1,4)}$ glycosidic bonds [12]; polysaccharide ring [13]; amino acids [14]; RSH-containing molecules [7]
1108	1099(s)	1100(s)	1099(s)	1100(s)	1104			1105(s)	ν_{as} (COC); ν (CC); ν (CO); δ (COH); ν $P(OH)_2$	$\beta_{(1,4)}$ glycosidic bonds [12]; amino acids [14]; polysaccharides [8,13]
1078	1078	1079	1075	1077	1073	1075	1063	1079	ν (CO); ν (CC); ν (COH); δ (COC); ϱ (NH_3^+); ν (SO)	Polysaccharides, proteins, and polyesters [15]; amino acid residues [16]
1044	1045	1046	1045	1044	1042	1042		1043	ν (CC); ν (CO); δ (COH); ν (SO)	Polysaccharides [9]; RSO_2H-containing molecules [7]
	1033(s)								ν (PO); ν (SH); ν (SO); backbone vibration	Polysaccharides and nucleic acids [2–4,8]
994	997	993	990		994		993	993	δ (NH_2); ν (CC); δ (HNCC); ν (CO); ν_s (PO_3^{2-}); δ (COH)	Amino acids [14,17]; $\beta_{(1,3)}$ glycosidic bonds [12]; nucleic acids [16]; polysaccharides [13]; RS^--containing molecules [7]

941	936	938	935	939	940		937	935	δ (=CH);<br>τ (CH_2);<br>ν_s (PO_4^{3-});<br>δ (SH); ν (SO);<br>δ (NH_2);<br>δ (CH_2)	Alkyl halides, carboxylic acids, amines, $\alpha_{(1,3)}$ glycosidic bonds [12]; amino acid residues [14]; nucleic acids [8]; RSH-, RSSR-, RSOSR-, and RSO_2SR-containing molecules [7,14]
917	916	913	916	917	916		915	915	δ (=CH);<br>τ (CH_2);<br>ν_s (PO_4^{3-});<br>δ (SH); ν (SO);<br>δ (NH_2);<br>δ (CH_2)	Alkyl halides, carboxylic acids; amines; $\alpha_{(1,3)}$ glycosidic bonds [12]; amino acid residues [14]; nucleic acids [8]; RSH-, RSSR-, RSOSR-, and RSO_2SR-containing molecules [7]
861	861	860	862	860	862	860	861	859	δ (NH_2); ν (CC);<br>ν (CN)	Amino acid residues [14]; RSO_2H-containing molecules [7]
805									ν SO(H); ν (PO)<br>δ (OH)<br>δ_{op} (HOCC)	Amino acids [14]; nucleic acids [18]
780	780	780	778	780	779	778	780	780	ν C(COOH);<br>δ (NH_2);<br>ϱ (CH_2);<br>δ (HNC);<br>δ (CCH);<br>δ (OH)	Amino acids [14]; RSH-, RSSR-, RSO_3^--, RSOSR-, and RSO_2SR-containing molecules [7]

Where ν, δ, β, ϱ, τ, and ω indicate stretching, bending, deformation, rocking, twisting, and wagging, respectively; sciss, oph, as, s, op, and ip stand for scissoring, out of phase, asymmetric, symmetric, out of plane and in plane vibrations.

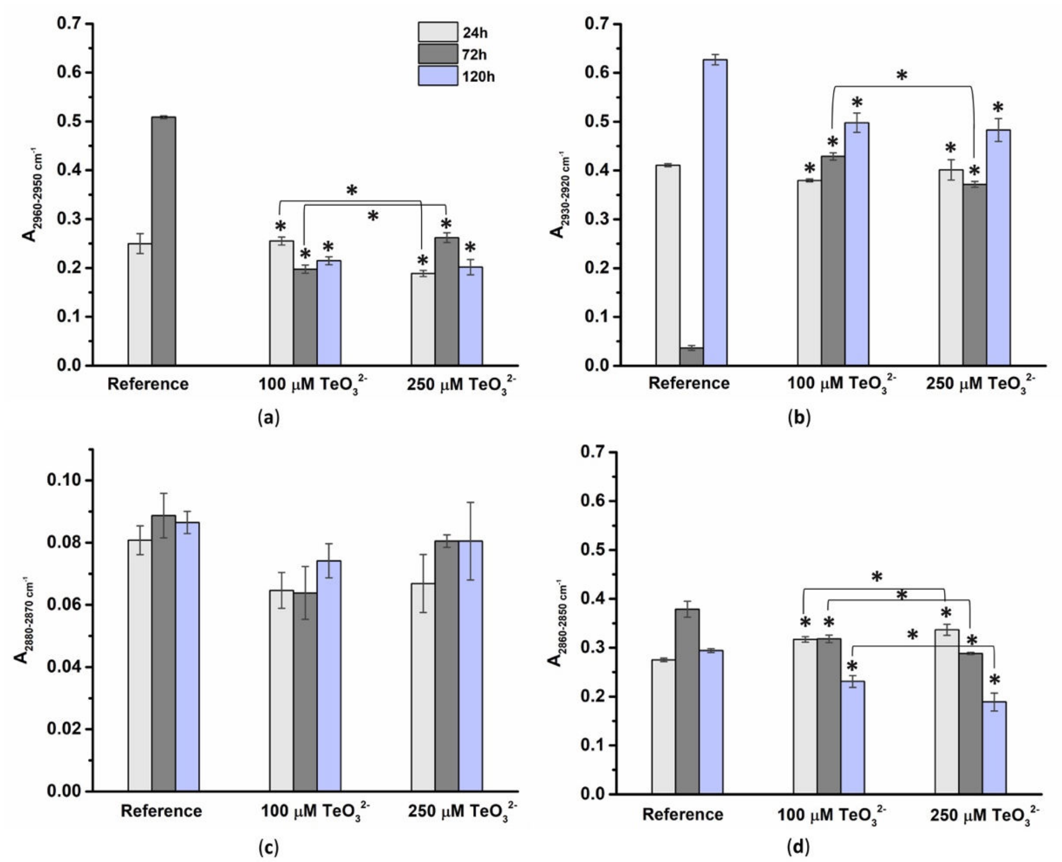

Figure S2. Normalized integrals deriving from ATR-FTIR contributions of (**a**) asymmetric -CH_3 (2960-2950 cm^{-1}), (**b**) asymmetric -CH_2 (2930-2920 cm^{-1}), (**c**) symmetric -CH (2880-2870 cm^{-1}), and (**d**) symmetric -CH_2 (2860-2850 cm^{-1}) vibrations typical of cellular lipids observed for *Micromonospora* cells grown in the absence or presence of TeO_3^{2-}. The symbol * indicates the statistical significance ($p < 0.05$) of the obtained integral variations.

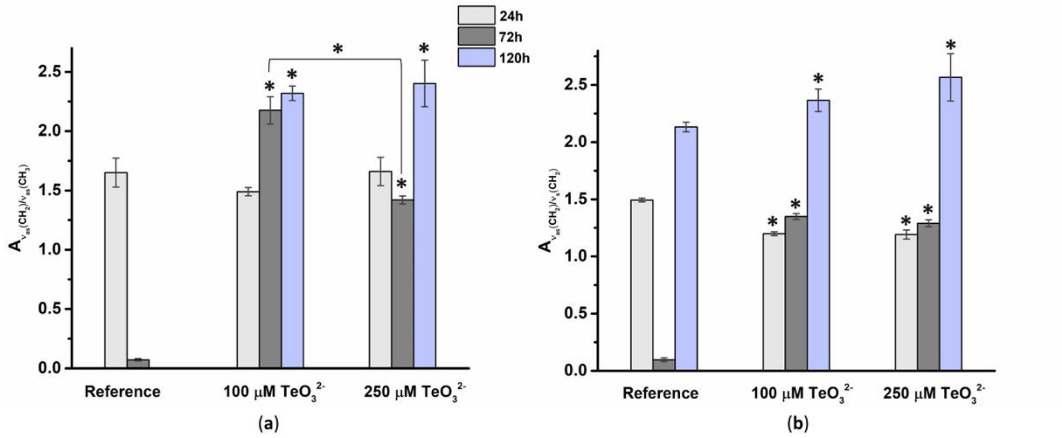

Figure S3. Ratios of normalized integrals deriving from ATR-FTIR contributions of (**a**) asymmetric -CH_2 (2930-2920 cm^{-1}) and asymmetric CH_3 (2960-2950 cm^{-1}) and (**b**) asymmetric -CH_2 (2930-2920 cm^{-1}) and symmetric -CH_2 (2860-2850 cm^{-1}) stretching vibrations. The symbol * indicates the statistical significance ($p < 0.05$) of the obtained integral variations.

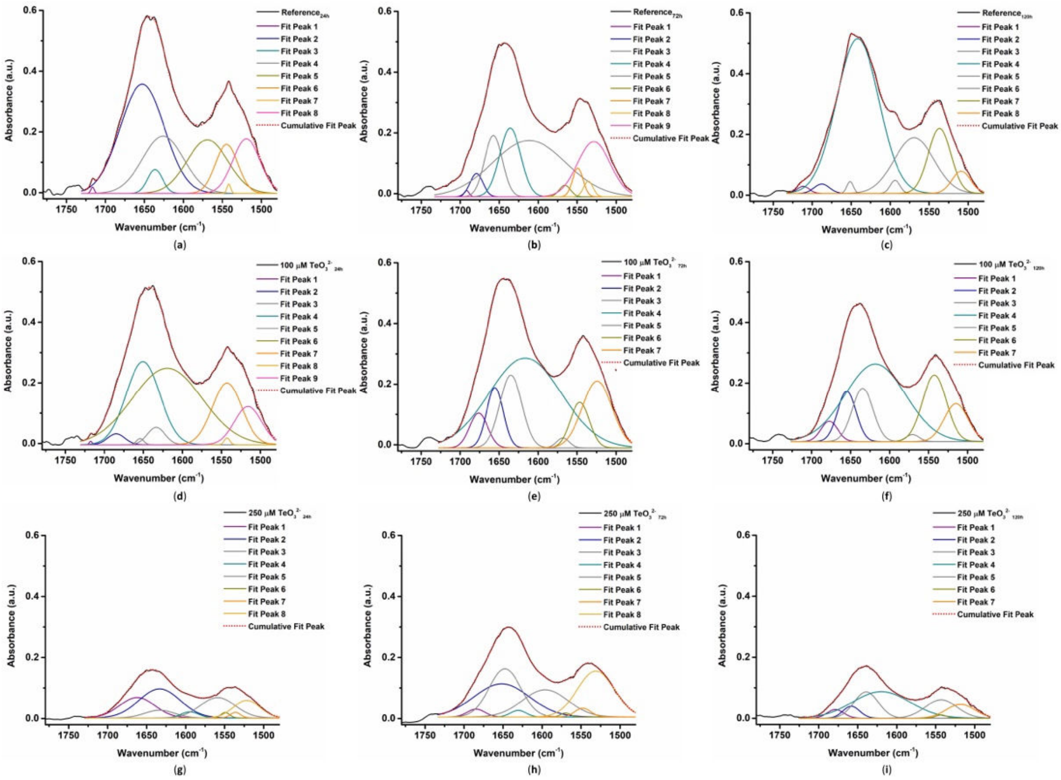

Figure S4. Deconvolution in the 1780-1480 cm^{-1} region of ATR-FTIR spectra obtained for *Micromonospora* cells grown in R5 liquid rich medium for (**a**) 24, (**b**) 72, and (**c**) 120-h, in R5 medium supplied with 100 µM TeO_3^{2-} for (**d**) 24, (**e**) 72, and (**f**) 120-h, or in R5 medium supplied with 250 µM TeO_3^{2-} for (**g**) 24, (**h**) 72, and (**i**) 120-h.

Table S2. Deconvolution of ATR-FTIR spectra of *Micromonospora* unchallenged cells in the 1780-1480 cm^{-1} region.

$Reference_{24h}$	w	A	$Reference_{72h}$	w	A	$Reference_{120h}$	w	A	Vibrational mode
1717	4.34 ± 0.46	0.11 ± 0.02				1712	15.7 ± 1.7	0.46 ± 0.03	ν (CO) [5]
			1692	6.45 ± 0.91	0.17 ± 0.02	1688	19.8 ± 2.3	0.77 ± 0.04	ν (CO); δ (NH) [2–4]
			1679	16.5 ± 1.7	1.58 ± 0.22				δ (NH); ν (CO) [5]
1653	56.5 ± 1.4	25.4 ± 0.8	1657	22.7 ± 2.3	5.74 ± 0.48	1651	8.36 ± 0.47	0.41 ± 0.02	ν (CO) [2–4]
1636	19.0 ± 1.5	1.88 ± 0.51	1635	27.0 ± 2.1	7.66 ± 1.4	1641	54.1 ± 1.1	34.6 ± 0.8	ν (CO); δ (NH) [5]
1626	52.6 ± 5.3	12.5 ± 0.8							ν (CO); δ (NH) [2]
			1612	93.7 ± 5.3	21.8 ± 1.4				ν (CO); δ (NH) [2]
						1594	12.6 ± 0.8	0.68 ± 0.09	$δ_{as}$ (NH_3^+) [3]
1569	52.4 ± 1.6	11.6 ± 0.9	1565	14.4 ± 1.3	0.69 ± 0.05	1569	50.8 ± 6.1	11.7 ± 1.2	$ν_{as}$ (COO^-) [2]
1544	29.0 ± 1.8	5.85 ± 0.32	1549	13.5 ± 1.7	1.60 ± 0.27				$δ_s$ (NH); ν (CN) [2–4]
1541	4.85 ± 0.56	0.19 ± 0.03	1536	12.0 ± 1.3	0.77 ± 0.06	1537	29.1 ± 1.9	7.81 ± 1.7	$δ_s$ (NH); ν (CN) [2–4]
			1528	41.9 ± 2.4	9.53 ± 0.92				$δ_s$ (NH); ν (CN) [2–4]
1519	34.7 ± 1.4	7.83 ± 0.92							$δ_s$ (NH_3^+) [3,7]
						1509	24.1 ± 1.3	2.22 ± 0.33	$δ_s$ (NH_3^+) [3,7]

where ν, δ, as, and s indicates stretching, bending, asymmetric, and symmetric vibrations, respectively.

Table S3. Deconvolution of ATR-FTIR spectra of *Micromonospora* cells challenged with 100 μM TeO_3^{2-} in the 1780-1480 cm^{-1} region.

100 μM $TeO_3^{2-}$$_{24h}$	w	A	100 μM $TeO_3^{2-}$$_{72h}$	w	A	100 μM $TeO_3^{2-}$$_{120h}$	w	A	Vibrational mode
1717	3.78 ± 0.82	0.54 ± 0.01							ν (CO) [5]
1685	22.1 ± 1.9	0.99 ± 0.08							ν (CO); δ (NH) [2–4]
			1676	23.0 ± 1.8	3.30 ± 0.54	1677	21.2 ± 2.6	1.78 ± 0.08	δ (NH); ν (CO) [5]
1654	9.29 ± 0.56	0.24 ± 0.02	1656	21.3 ± 1.8	5.29 ± 0.48	1654	23.5 ± 2.2	4.88 ± 0.32	ν (CO) [2–4]
1650	43.3 ± 2.8	14.9 ± 1.3							ν (CO); δ (NH) [2–4]
1633	22.6 ± 0.8	1.61 ± 0.21	1634	28.3 ± 2.2	8.49 ± 1.67	1635	26.6 ± 2.6	5.85 ± 0.44	ν (CO); δ (NH) [5]
1619	89.3 ± 6.5	28.2 ± 2.5	1616	92.8 ± 2.8	34.4 ± 0.7	1618	77.5 ± 2.7	24.8 ± 0.9	ν (CO); δ (NH) [2]
			1569	16.1 ± 1.9	0.65 ± 0.12	1570	17.0 ± 1.8	0.49 ± 0.03	$ν_{as}$ (COO^-) [2]
1544	38.0 ± 1.2	9.68 ± 0.82	1546	24.3 ± 1.6	4.56 ± 0.72	1542	30.8 ± 2.4	8.46 ± 1.02	$δ_s$ (NH); ν (CN) [2–4]
1540	6.97 ± 0.56	0.19 ± 0.02				1537	29.1 ± 1.9	7.81 ± 0.92	$δ_s$ (NH); ν (CN) [2–4]
			1524	38.3 ± 1.9	10.5 ± 1.2				$δ_s$ (NH); ν (CN) [2–4]
1515	34.8 ± 3.7	5.48 ± 0.59				1515	30.4 ± 3.1	4.75 ± 0.55	$δ_s$ (NH_3^+) [3,7]

Where ν, δ, as, and s indicates stretching, bending, asymmetric, and symmetric vibrations, respectively.

Table S4. Deconvolution of ATR-FTIR spectra of *Micromonospora* cells challenged with 250 μM TeO_3^{2-} in the 1780-1480 cm^{-1} region.

250 μM TeO_3^{2-} 24h	w	A	250 μM TeO_3^{2-} 72h	w	A	250 μM TeO_3^{2-} 120h	w	A	Vibrational mode
			1684	22.7 ± 1.7	0.73 ± 0.09				ν (CO); δ (NH) [2–4]
						1679	21.4 ± 2.1	0.77 ± 0.05	δ (NH); ν (CO) [5]
1662	45.2 ± 5.1	3.79 ± 0.54	1652	71.3 ± 4.4	9.70 ± 0.98	1658	21.5 ± 1.8	1.05 ± 0.04	ν (CO) [2–4]
			1647	40.3 ± 3.8	8.00 ± 0.55				ν (CO); δ (NH) [2–4]
1633	52.9 ± 1.5	6.30 ± 0.65	1630	20.2 ± 1.9	8.55 ± 0.42	1639	31.3 ± 2.7	3.38 ± 0.28	ν (CO); δ (NH) [5]
1628	38.3 ± 3.9	1.22 ± 0.11							ν (CO); δ (NH) [2]
						1619	73.9 ± 2.6	8.06 ± 0.88	ν (CO); δ (NH) [2]
			1596	58.3 ± 4.1	6.52 ± 0.71				δ_{as} (NH_3^+) [3]
			1569	12.4 ± 1.1	0.20 ± 0.02				ν_{as} (COO^-) [2]
1559	45.0 ± 3.8	3.78 ± 0.33							δ_s (NH); ν (CN) [2–4]
1549	11.5 ± 1.2	0.26 ± 0.02	1547	20.7 ± 1.8	0.74 ± 0.09	1542	41.0 ± 3.2	3.10 ± 0.41	δ_s (NH); ν (CN) [2–4]
1536	12.9 ± 1.3	0.30 ± 0.02	1531	47.0 ± 5.1	8.88 ± 0.91	1539	13.6 ± 1.3	0.18 ± 0.08	δ_s (NH); ν (CN) [2–4]
1521	34.6 ± 1.4	2.49 ± 0.22							δ_s (NH); ν (CN) [2–4]
						1518	36.9 ± 1.5	2.11 ± 0.34	δ_s (NH_3^+) [3,7]

Where ν, δ, as, and s indicates stretching, bending, asymmetric, and symmetric vibrations, respectively.

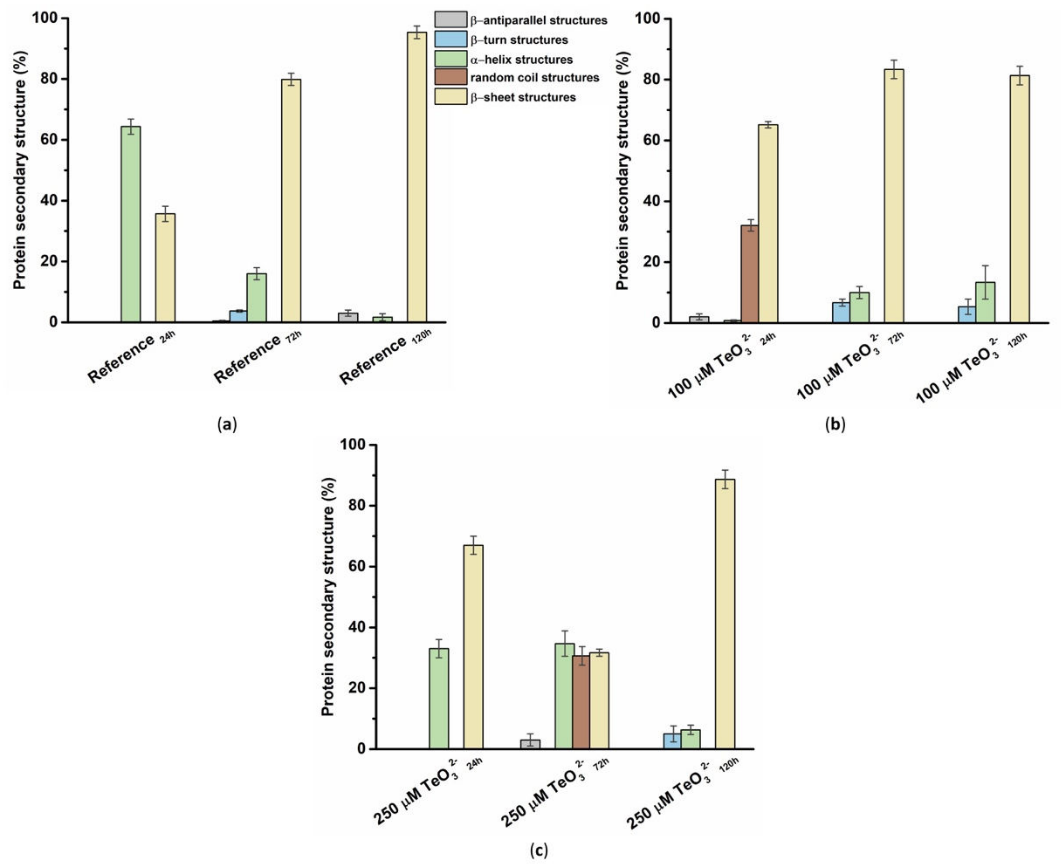

Figure S5. Estimation of protein secondary structures derived from normalized integrals of amide I bands of ATR-FTIR spectra obtained for *Micromonospora* cells grown in (**a**) R5 liquid rich medium or R5 medium supplied with (**b)** 100 μM TeO_3^{2-} or (**c**) 250 μM TeO_3^{2-}.

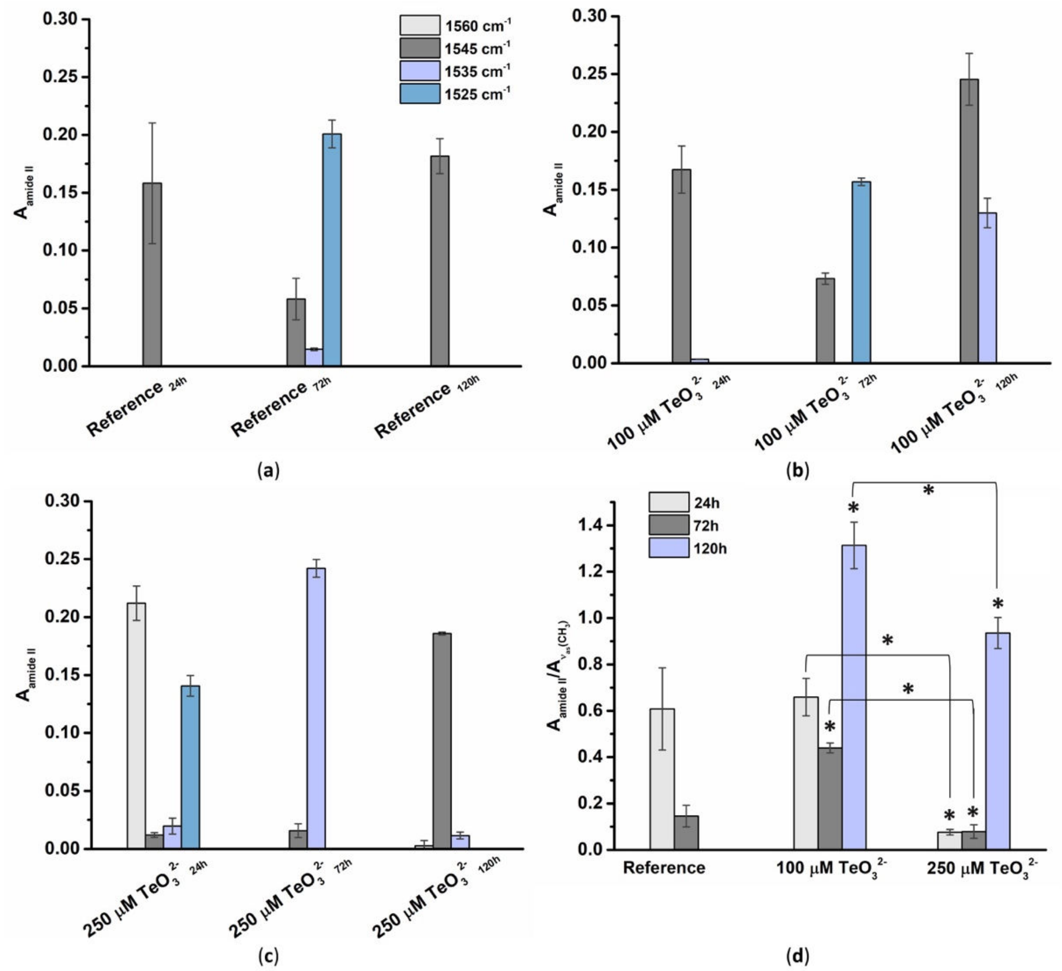

Figure S6. Distribution of normalized integrals referring to amide II bands of ATR-FTIR spectra obtained for *Micromonospora* cells grown in (**a**) R5 liquid rich medium or R5 medium supplied with (**b)** 100 μM TeO_3^{2-} or (**c**) 250 μM TeO_3^{2-}. (**d**) Ratios of normalized integrals deriving from ATR-FTIR contributions of amide II bands (1560-1520 cm^{-1}) and asymmetric CH_3 stretching vibrations (2960-2950 cm^{-1}). The symbol * indicates the statistical significance ($p < 0.05$) of the obtained integral variations.

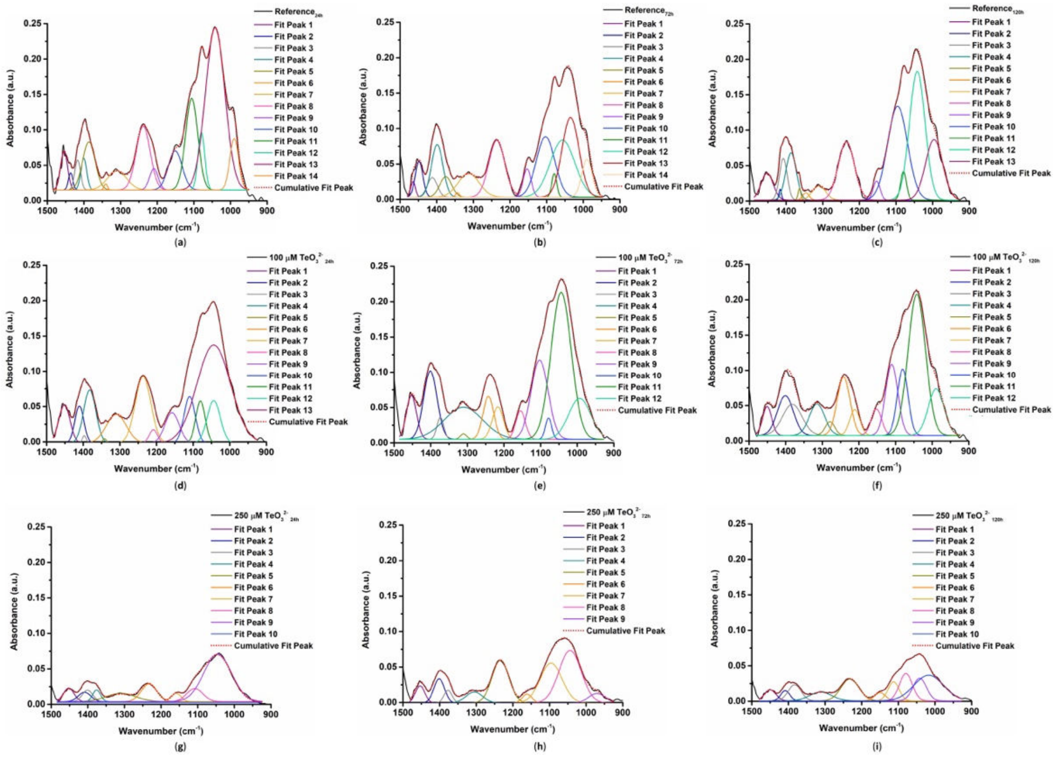

Figure S7. Deconvolution in the 1500-900 cm^{-1} region of ATR-FTIR spectra obtained for *Micromonospora* cells grown in R5 liquid rich medium for (**a**) 24, (**b**) 72, and (**c**) 120-h, in R5 medium supplied with 100 μM TeO_3^{2-} for (**d**) 24, (**e**) 72, and (**f**) 120-h, or in R5 medium supplied with 250 μM TeO_3^{2-} for (**g**) 24, (**h**) 72, and (**i**) 120-h.

Table S5. Deconvolution of ATR-FTIR spectra of *Micromonospora* unchallenged cells in the 1500-800 cm^{-1} region.

Reference$_{24h}$	w	A	Reference$_{72h}$	w	A	Reference$_{120h}$	w	A	Vibrational mode
			1465	12.7 ± 1.6	0.35 ± 0.02				δ (CH$_2$); δ (CH$_3$) [4]
1454	20.8 ± 0.9	1.51 ± 0.05	1448	20.8 ± 1.9	1.32 ± 0.05	1453	32.1 ± 1.1	1.45 ± 0.06	δ_{sciss} (CH$_2$); δ (OH); ν CC(O); ν_s (COO$^-$) [7–9]
1436	11.4 ± 1.3	0.31 ± 0.03							ν_s (COO$^-$) [7–9]
1417	16.3 ± 1.0	0.90 ± 0.06	1412	27.2 ± 1.1	0.94 ± 0.03	1416	8.19 ± 0.9	0.20 ± 0.01	ν_s (COO$^-$) [5]
1400	15.7 ± 0.8	0.86 ± 0.07	1398	31.3 ± 1.7	2.92 ± 0.22	1404	18.5 ± 1.1	0.76 ± 0.05	δ (CH); δ (OH); δ (COH); β (CH$_3$); ν (CN); ν_s (COO$^-$) [5]
1387	35.0 ± 3.2	3.12 ± 0.12				1391	36.5 ± 2.4	3.23 ± 0.81	δ (CH); δ (COH); β (CH$_3$); ν CC(O); ν_s (COO$^-$); δ (NH$_2$); ν (CN) [9,10]
			1375	32.4 ± 3.4	1.18 ± 0.12				β (CH$_3$); ν_s (COO$^-$) [4,7]
						1364	5.48 ± 0.67	0.08 ± 0.02	β (CH$_3$); δ (CH); δ (OH); ν_s (COO$^-$) [4,7]
1341	8.94 ± 0.88	0.10 ± 0.02	1343	10.4 ± 0.9	0.08 ± 0.01	1341	0.05 ± 0.01	49.4 ± 3.2	β C(OH); δ (OH); δ (CH) [4,8]
1310	56.3 ± 5.1	2.14 ± 0.17	1313	57.6 ± 1.4	2.42 ± 0.45	1314	45.6 ± 4.0	1.02 ± 0.21	ν C-(OH) [11]
1239	38.0 ± 2.0	4.41 ± 0.38	1236	43.5 ± 1.0	4.38 ± 0.19	1235	42.4 ± 2.5	4.33 ± 0.38	β (NH); ν_{as} (PO$_2^-$) [2]
1209	24.2 ± 1.8	1.00 ± 0.33							ν_s (PO$_2^-$) [7]
1150	39.8 ± 1.9	2.94 ± 0.03	1153	21.6 ± 1.1	1.086 ± 0.12	1152	18.4 ± 0.9	0.61 ± 0.02	ν_{as} (COC); δ (CH$_2$); δ (CH); δ (NH$_2$) [12–14]
1106	39.8 ±1.6	5.43 ± 0.56	1102	50.2 ± 4.9	5.38 ± 0.43	1097	52.7 ± 3.6	8.50 ± 0.91	ν_{as} (COC); ν (CC); ν (CO); δ (COH); ν P(OH)$_2$ [5,8,12,14]
1079	18.7 ± 1.4	2.00 ± 0.18	1079	13.4 ± 0.8	0.56 ± 0.07	1079	16.1 ± 1.2	0.82 ± 0.07	ν (CO); ν (CC); ν (COH); δ (COC); ϱ (NH$_3^+$) [15,16]
			1057	67.1 ± 3.2	6.78 ± 0.92				ν (CC); ν (CO); δ (COH); ν_s (PO$_2^-$) [7,14]
1041	52.1 ± 4.9	15.2 ± 1.1	1035	46.3 ± 3.8	6.54 ± 0.52	1041	44.2 ± 2.8	10.5 ± 1.2	ν (CO); ν (PO) [2–4,8]
990	25.6 ± 1.9	2.43 ± 0.32	990	28.8 ± 2.1	1.93 ± 0.22	994	43.7 ± 3.5	4.35 ± 0.56	δ (NH$_2$); δ (HNCC); ν (CO); ν_s (PO$_2^-$); ν (CO); ν (CC) [5,7,12,14,16,17]

where ν, δ, β, ϱ, and ω indicate stretching, bending, deformation, rocking, and wagging, respectively; sciss, oph, as, s, and ip stand for scissoring, out of phase, asymmetric, symmetric, and in plane vibrations.

Table S6. Deconvolution of ATR-FTIR spectra of *Micromonospora* cells challenged with 0.1 mM TeO_3^{2-} in the 1500-800 cm^{-1} region.

100 μM TeO_3^{2-} 24h	w	A	100 μM TeO_3^{2-} 72h	w	A	100 μM TeO_3^{2-} 120h	w	A	Vibrational mode
1451	30.5 ± 0.9	2.08 ± 0.11	1454	23.8 ± 0.6	1.50 ± 0.07	1451	24.9 ± 2.3	1.27 ± 0.08	δ_{sciss} (CH_2); δ (OH); ν CC(O); ν_s (COO^-) [7–9]
1411	24.6 ± 2.1	1.61 ± 0.22							ν_s (COO^-) [5]
1400	15.7 ± 2.1	0.86 ± 0.08	1401	56.4 ± 2.1	4.09 ± 0.20	1401	43.6 ± 1.9	3.07 ± 0.03	δ (CH); δ (OH); δ (COH); β (CH_3); ν (CN); ν_s (COO^-) [5]
1382	33.4 ± 1.7	3.14 ± 0.29				1382	53.3 ± 1.6	2.95 ± 0.33	δ (CH); δ (COH); β (CH_3); ν CC(O); ν_s (COO^-); δ (NH_2); ν (CN) [9,10]
			1375	20.4 ± 1.0	0.91 ± 0.05				β (CH_3); ν_s (COO^-) [4,7]
1341	10.9 ± 1.8	0.09 ± 0.01							β C(OH); δ (OH); δ (CH) [4,7,8]
1310	54.6 ± 2.9	2.80 ± 0.18	1310	56.4 ± 3.5	2.71 ± 0.29				ν C-(OH) [11]
			1305	10.1 ± 1.3	1.22 ± 0.05	1304	41.8 ± 2.4	2.33 ± 0.13	ν (C-OH) [11]
						1283	26.4 ± 1.1	0.64 ± 0.03	ν (C-OH) [11]
1237	42.5 ± 1.6	5.01 ± 0.26	1238	37.5 ± 1.7	3.86 ± 0.31	1242	35.3 ± 3.9	3.66 ± 0.24	β (NH); ν_{as} (PO_2^-) [2]
1208	22.1 ± 2.2	0.53 ± 0.06	1210	25.4 ± 1.8	0.76 ± 0.03	1211	27.8 ± 1.8	1.27 ± 0.04	ν_s (PO_2^-) [7]
1157	38.9 ± 3.3	2.10 ± 0.24	1153	26.5 ± 0.9	1.35 ± 0.09	1152	28.5 ± 2.9	1.33 ± 0.21	ν_{as} (COC); δ (CH_2); δ (CH); δ (NH_2) [5,7,12,14]
1109	30.5 ± 3.1	2.51 ± 0.19	1104	43.1 ± 2.7	5.87 ± 0.61	1110	33.6 ± 2.5	4.26 ± 0.16	ν_{as} (COC); ν (CC); ν (CO); δ (COH); ν $P(OH)_2$ [8,12–14]
1080	25.7 ± 2.2	1.92 ± 0.23	1078	19.1 ± 1.1	0.88 ± 0.07	1081	29.4 ± 2.2	3.46 ± 0.41	ν (CO); ν (CC); ν (COH); δ (COC); ϱ (NH_3^+) [15,16]
1044	35.7 ± 3.8	2.69 ± 0.36	1046	53.7 ± 3.1	12.9 ± 1.4	1042	47.5 ± 3.8	11.9 ± 1.2	ν (CO); ν (PO); ν (SH); ν (SO) [7,14]
1039	91.4 ± 5.1	15.9 ± 1.1							ν (PO); ν (SH); ν (SO); backbone vibration [2–4,7,8]
			1002	64.6 ± 6.1	5.69 ± 0.62				δ (NH_2); δ (HNCC); ν (CO); ν_s (PO_3^{2-}) [7]
						989	42.9 ± 2.6	3.54 ± 0.37	δ (NH_2); δ (HNCC); ν (CO); ν_s (PO_2^-); ν (CO); ν (CC) [5,7,12,14,16,17]

Where ν, δ, β, ϱ, and ω indicate stretching, bending, deformation, rocking, and wagging, respectively; sciss, oph, as, s, and ip stand for scissoring, out of phase, asymmetric, symmetric, and in plane vibrations.

Table S7. Deconvolution of ATR-FTIR spectra of *Micromonospora* cells challenged with 0.25 mM TeO_3^{2-} in the 1500-800 cm^{-1} region.

250 μM $TeO_3^{2-}$$_{24h}$	w	A	250 μM $TeO_3^{2-}$$_{72h}$	w	A	250 μM $TeO_3^{2-}$$_{120h}$	w	A	Vibrational mode
1452	28.9 ± 2.1	0.69 ± 0.04	1453	26.5 ± 2.0	0.77 ± 0.06	1450	31.3 ± 1.2	0.61 ± 0.04	δ_{sciss} (CH_2); δ (OH); ν CC(O); ν_s (COO^-) [7–9]
1409	34.0 ± 2.4	0.56 ± 0.03				1409	24.8 ± 1.8	0.45 ± 0.05	ν_s (COO^-) [5]
1400	28.7 ± 1.1	0.58 ± 0.05	1401	28.8 ± 2.6	1.23 ± 0.22				δ (CH); δ (OH); δ (COH); β (CH_3); ν (CN); ν_s (COO^-) [5]
						1383	35.3 ± 2.5	1.01 ± 0.10	δ (CH); δ (COH); β (CH_3); ν CC(O); ν_s (COO^-); δ (NH_2); ν (CN); ν_s (COO^-) [9,10]
1375	21.3 ± 1.7	0.44 ± 0.03	1377	21.8 ± 1.9	0.49 ± 0.05				β (CH_3); ν_s (COO^-) [4,7]
1306	80.3 ± 1.5	1.18 ± 0.10	1305	45.3 ± 4.0	3.28 ± 0.37	1302	60.7 ± 2.4	0.95 ± 0.06	ν (C-OH) [11]
1232	42.4 ± 1.8	1.33 ± 0.12	1233	46.0 ± 0.4	3.49 ± 0.08	1232	48.9 ± 0.7	1.90 ± 0.05	β (NH); ν_{as} (PO_2^-) [2]
1159	26.5 ± 1.9	0.39 ± 0.06	1161	25.9 ± 1.3	0.40 ± 0.02	1150	29.9 ± 2.2	0.44 ± 0.04	ν_{as} (COC); δ (CH_2); δ (CH); δ (NH_2) [5,7,12,14]
1108	44.0 ± 2.3	1.02 ± 0.11	1095	59.6 ± 3.9	4.19 ± 0.52	1113	30.3 ± 3.3	1.02 ± 0.21	ν_{as} (COC); ν (CC); ν (CO); δ (COH); ν $P(OH)_2$ [8,12–14]
						1079	35.3 ± 5.3	1.71 ± 0.19	ν (CO); ν (CC); ν (COH); δ (COC); ϱ (NH_3^+) [15,16]
1045	75.6 ± 2.1	6.33 ± 0.37	1044	60.5 ± 3.2	5.62 ± 0.51	1042	36.8 ± 3.7	1.48 ± 0.18	ν (CO); ν (PO); ν (SH); ν (SO) [7,14]
						1017	81.1 ± 4.3	3.68 ± 0.24	
			970	44.6 ± 3.9	0.74 ± 0.02				δ (NH_2); δ (HNCC); ν (CO); ν_s (PO_2^-); ν (CO); ν (CC) [5,7,12,14,16,17]

Where ν, δ, β, ϱ, and ω indicate stretching, bending, deformation, rocking, and wagging, respectively; sciss, oph, as, s, and ip stand for scissoring, out of phase, asymmetric, symmetric, and in plane vibrations.

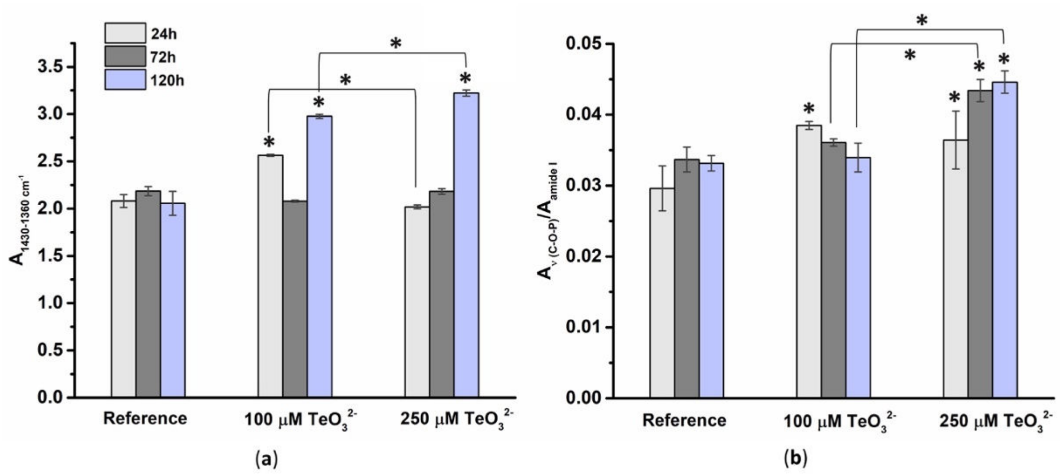

Figure S8. Normalized integrals referring to (**a**) ATR-FTIR contributions attributable to peroxidation products (1430-1360 cm-1) and (**b**) ratios deriving from IR signals related to the -C-O-P stretching vibration (1235 cm-1) and the amide I (1690-1610 cm-1) band. The symbol * indicates the statistical significance ($p < 0.05$) of the obtained integral variations.

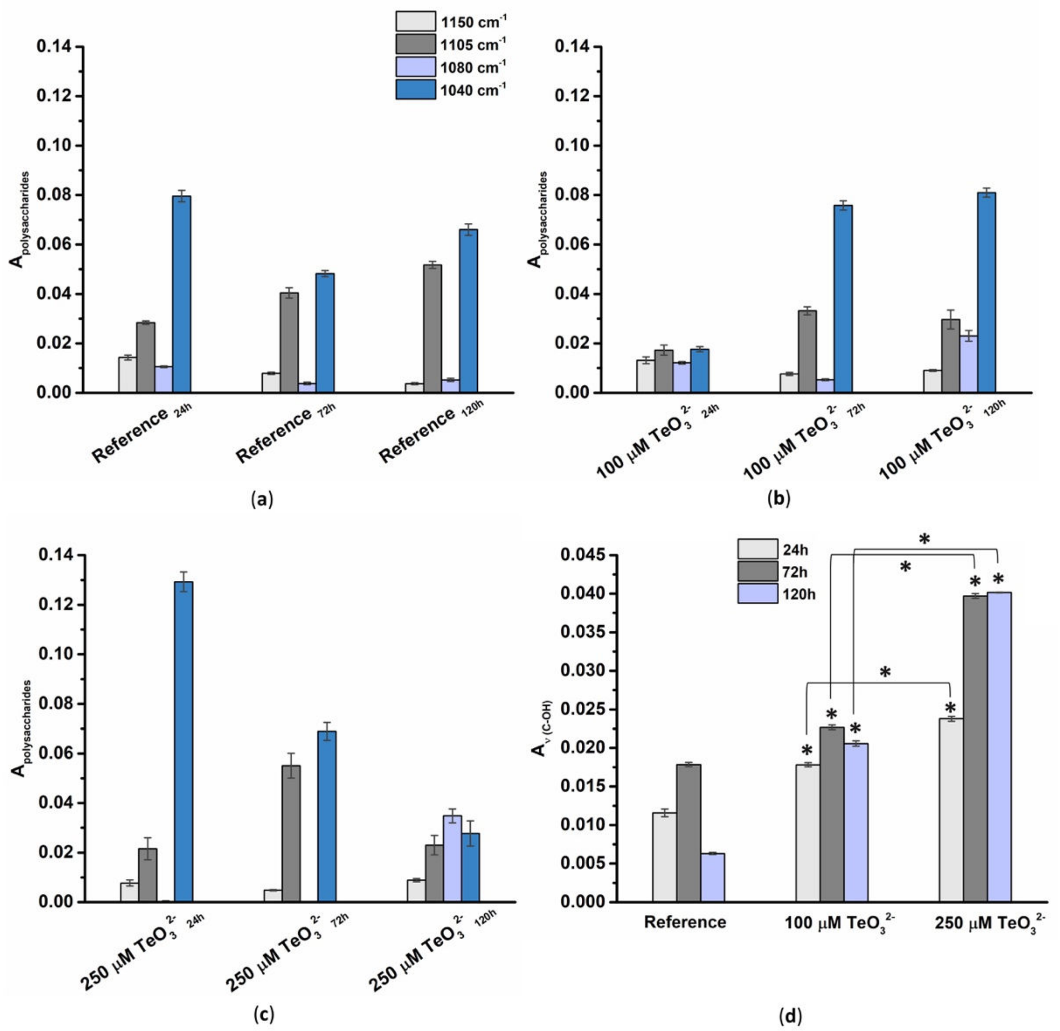

Figure S9. Distribution of normalized integrals referring to polysaccharide vibrations of ATR-FTIR spectra obtained for *Micromonospora* cells grown in (**a**) R5 liquid-rich medium or R5 medium supplied with (**b**) 100 μM TeO_3^{2-} or (**c**) 250 μM TeO_3^{2-}. In (d) are depicted the normalized integrals referring to ATR-FTIR contributions of -C-(OH) stretching (1310-1300 cm-1). The symbol * indicates the statistical significance ($p < 0.05$) of the obtained integral variations.

References

1. Zonaro, E.; Piacenza, E.; Presentato, A.; Monti, F.; Dell'Anna, R.; Lampis, S.; Vallini, G. Ochrobactrum Sp. MPV1 from a Dump of Roasted Pyrites Can Be Exploited as Bacterial Catalyst for the Biogenesis of Selenium and Tellurium Nanoparticles. *Microb. Cell Fact.* **2017**, *16*, 1–17, doi:https://doi.org/10.1186/s12934-017-0826-2.
2. Tugarova, A.V.; Mamchenkova, P.V.; Dyatlova, Y.A.; Kamnev, A.A. FTIR and Raman Spectroscopic Studies of Selenium Nanoparticles Synthesised by the Bacterium Azospirillum Thiophilum. *Spectrochim. Acta A Mol. Biomol. Spectrosc.* **2018**, *192*, 458–463, doi:https://doi.org/10.1016/j.saa.2017.11.050.
3. Faghihzadeh, F.; Anaya, N.M.; Schifman, L.A.; Oyanedel-Craver, V. Fourier Transform Infrared Spectroscopy to Assess Molecular-Level Changes in Microorganisms Exposed to Nanoparticles. *Nanotechnol. Environ. Eng.* **2016**, *1*, 1–16, doi:https://doi.org/10.1007/s41204-016-0001-8.
4. Lasch, P.; Naumann, D. Infrared Spectroscopy in Microbiology. In *Encyclopedia of Analytical Chemistry*; John Wiley & Sons, Ltd, 2015; pp. 1–32.
5. Kiwi, J.; Nadtochenko, V. Evidence for the Mechanism of Photocatalytic Degradation of the Bacterial Wall Membrane at the TiO 2 Interface by ATR-FTIR and Laser Kinetic Spectroscopy. *Langmuir* **2005**, *21*, 4631–4641, doi:https://doi.org/10.1021/la046983l.
6. Wang, X.; Wang, W.; Liu, P.; Wang, P.; Zhang, L. Photocatalytic Degradation of E.Coli Membrane Cell in the Presence of ZnO Nanowires. *J. Wuhan Univ. Technol. Mater. Sci. Ed.* **2011**, *26*, 222–225, doi:10.1007/S11595-011-0201-9.
7. Piacenza, E.; Presentato, A.; Ferrante, F.; Cavallaro, G.; Alduina, R.; Chillura Martino, D.F. Biogenic Selenium Nanoparticles: A Fine Characterization to Unveil Their Thermodynamic Stability. *Nanomaterials* **2021**, *11*, 1195, doi:https://doi.org/10.3390/nano11051195.
8. Jiang, W.; Saxena, A.; Song, B.; Ward, B.B.; Beveridge, T.J.; Myneni, S.C.B. Elucidation of Functional Groups on Gram-Positive and Gram-Negative Bacterial Surfaces Using Infrared Spectroscopy. *Langmuir* **2004**, *20*, 11433–11442, doi:https://doi.org/10.1021/la049043+.
9. Buszewski, B.; Dziubakiewicz, E.; Pomastowski, P.; Hrynkiewicz, K.; Ploszaj-Pyrek, J.; Talik, E.; Kramer, M.; Albert, K. Assignment of Functional Groups in Gram-Positive Bacteria. *J. Anal. Bioanal. Tech.* **2015**, *6*, 1–8, doi:https://doi.org/10.4172/2155-9872.1000232.
10. Otari, S.V.; Patil, R.M.; Ghosh, S.J.; Thorat, N.D.; Pawar, S.H. Intracellular Synthesis of Silver Nanoparticle by Actinobacteria and Its Antimicrobial Activity. *Spectrochim. Acta A Mol. Biomol. Spectrosc.* **2015**, *136*, 1175–1180, doi:https://doi.org/10.1016/j.saa.2014.10.003.
11. Kepenek, E.S.; Gozen, A.G.; Severcan, F. Molecular Characterization of Acutely and Gradually Heavy Metal Acclimated Aquatic Bacteria by FTIR Spectraoscopy. *J. Biophotonics* **2019**, *12*, doi:https://doi.org/10.1002/jbio.201800301.
12. Nikonenko, N.A.; Buslov, D.K.; Sushko, N.I.; Zhbankov, R.G.; Stepanov, B.I. Investigation of Stretching Vibrations of Glycosidic Linkages in Disaccharides and Polysaccarides with Use of IR Spectra Deconvolution. *Biopolymers* **2000**, *57*, 257–262, doi:https://doi.org/10.1002/1097-0282(2000)57:4<257::AID-BIP7>3.0.CO;2-3.
13. Nadtochenko, V.A.; Rincon, A.G.; Stanca, S.E.; Kiwi, J. Dynamics of E. Coli Membrane Cell Peroxidation during TiO2 Photocatalysis Studied by ATR-FTIR Spectroscopy and AFM Microscopy. *J. Photochem. Photobiol. A* **2005**, *169*, 131–137, doi:https://doi.org/10.1016/j.jphotochem.2004.06.011.
14. Mohamed, M.E.; Mohammed, A.M.A. Experimental and Computational Vibration Study of Amino Acids. *Int. lett. chem. phys. astron.* **2013**, *15*, 1–17, doi:https://doi.org/10.18052/www.scipress.com/ILCPA.15.1.
15. Kamnev, A.A.; Mamchenkova, P. v.; Dyatlova, Y.A.; Tugarova, A. v.; Kamnev, A.A.; Mamchenkova, P. v.; Dyatlova, Y.A.; Tugarova, A. v. FTIR Spectroscopic Studies of Selenite Reduction by Cells of the Rhizobacterium Azospirillum Brasilense Sp7 and the Formation of Selenium Nanoparticles. *J. Mol. Struct.* **2017**, *1140*, 106–112, doi:https://doi.org/10.1016/j.molstruc.2016.12.003.

16. Kurihara, T.; Noda, Y.; Takegoshi, K. Capping Structure of Ligand-Cysteine on CdSe Magic-Sized Clusters. *ACS Omega* **2019**, *4*, 3476–3483, doi:https://doi.org/10.1021/acsomega.8b02752.
17. Barth, A. The Infrared Absorption of Amino Acid Side Chains. *Prog. Biophys. Mol. Biol.* **2000**, *74*, 141–173, doi:https://doi.org/10.1016/S0079-6107(00)00021-3.
18. Garip, S.; Gozen, A.C.; Severcan, F. Use of Fourier Transform Infrared Spectroscopy for Rapid Comparative Analysis of Bacillus and Micrococcus Isolates. *Food Chem.* **2009**, *113*, 1301–1307, doi:https://doi.org/10.1016/j.foodchem.2008.08.063.
